# EDEN: A High-Performance, General-Purpose, NeuroML-Based Neural Simulator

**DOI:** 10.3389/fninf.2022.724336

**Published:** 2022-05-20

**Authors:** Sotirios Panagiotou, Harry Sidiropoulos, Dimitrios Soudris, Mario Negrello, Christos Strydis

**Affiliations:** ^1^School of Electrical and Computer Engineering, National Technical University of Athens, Athens, Greece; ^2^Department of Neuroscience, Erasmus Medical Center, Rotterdam, Netherlands; ^3^Quantum and Computer Engineering Department, Delft University of Technology, Delft, Netherlands

**Keywords:** computational neuroscience, biological neural networks, simulation, High-Performance Computing, code morphing, interoperability, NeuroML, software

## Abstract

Modern neuroscience employs *in silico* experimentation on ever-increasing and more detailed neural networks. The high modeling detail goes hand in hand with the need for high model reproducibility, reusability and transparency. Besides, the size of the models and the long timescales under study mandate the use of a simulation system with high computational performance, so as to provide an acceptable time to result. In this work, we present EDEN (Extensible Dynamics Engine for Networks), a new general-purpose, NeuroML-based neural simulator that achieves both high model flexibility and high computational performance, through an innovative model-analysis and code-generation technique. The simulator runs NeuroML-v2 models directly, eliminating the need for users to learn yet another simulator-specific, model-specification language. EDEN's functional correctness and computational performance were assessed through NeuroML models available on the NeuroML-DB and Open Source Brain model repositories. In qualitative experiments, the results produced by EDEN were verified against the established NEURON simulator, for a wide range of models. At the same time, computational-performance benchmarks reveal that EDEN runs from one to nearly two orders-of-magnitude faster than NEURON on a typical desktop computer, and does so without additional effort from the user. Finally, and without added user effort, EDEN has been built from scratch to scale seamlessly over multiple CPUs and across computer clusters, when available.

## 1. Introduction

Simulation of biological neural networks is an essential tool of modern neuroscience. However, there are currently certain challenges associated with the development and *in silico* study of such networks. The neural models in use are diverse and heterogeneous; there is no single set of mathematical formulae that is commonly used by the majority of existing models. In addition, the biophysical mechanisms that make up models are constantly being modified, and reused in various combinations in new models. These factors mandate the use of general-purpose neural simulators in common practice. At the same time, the network sizes and levels of modeling detail employed in modern neuroscience translate to a constant increase in the volume of required computations. Thus, neuroscience projects necessitate high-performance tools for simulations to finish in a practical amount of time and for models to fit into available computer memory.

Although there already exists a rich arsenal of simulators targeting neuroscience, the aforementioned challenges of neural simulation remain an open problem. On one hand, there are hand-written codes that push the processing hardware to the limit but they are difficult or impossible to extend in terms of model support, because of their over-specialization. They offer great computational performance by executing solely the numerical calculations required by the model's dynamics. On the other hand, there are general-purpose simulators that readily support most types of models, however, their computational efficiency is much less than that of hand-written codes. Hence, there is a significant gap in efficiency between general-purpose neural simulators and the computational capabilities that modern hardware platforms can achieve.

Besides, simulation of large networks often requires deploying neural models on multiple processor cores or, even, on computer clusters. Existing general-purpose simulators do not manage the technicalities of parallelization, model decomposition, and communication automatically. Thus, significant engineering effort is spent on setting up the simulators to run on multi-core and multi-node systems, which further obstructs scientific work.

A further problem is that, presently, each neural simulator uses its own model-specification language. Thus, models written for one simulator are difficult and laborious to adapt for another, which hampers the exchange and reuse of models across the neuroscience community. In this context, if a new simulator were to support only its own modeling language, this would fragment the modeling community further and would add a serious barrier to the simulator's adoption as well as the reuse of existing models.

### 1.1. The EDEN Simulator

To address the challenges in *in silico* neuroscience, we designed a new general-purpose neural simulator, called EDEN (Extensible Dynamics Engine for Networks). EDEN directly runs models described in NeuroML, achieves leading computational performance through a novel architecture, and handles parallel-processing resources—both on standalone personal computers as well as on computer clusters—automatically.

EDEN employs an innovative *model-analysis* and *code-generation technique*^1^ through which the model's variables and the mathematical operations needed for the simulation are converted into a set of individual *work items*. Each work item consists of the data that represent a part of the neural network, and the calculations to simulate this part of the network over time. The calculations for the individual work items can then be run *in parallel* within each simulation step, allowing distribution of the computational load among many processing elements. This technique enables *by-design support* for general neural models, and at the same time offers significant performance benefits over conventional approaches. The need for model generality with user-provided formulae is *directly* addressed *via automatic code generation*; but the architecture also supports hand-optimized implementations that apply for specific types of neurons. At the same time, reducing the complex structure of biophysical mechanisms inside a neuron into an explicitly laid out set of essential, model-specific calculations allows compilers to perform large-scale optimizations. What is more, traditional simulators perform best with specific kinds of neuron models (e.g., multi-compartmental or point neurons) and worse with other ones. In contrast, EDEN's approach allows selecting the implementation that works best for each part of the network, at run time.

We adopted the *NeuroML v2* standard (Cannon et al., 2014) as our simulator's modeling language. NeuroML v2 is the emerging, standard cross-tool specification language for general neural-network models. By following the standard, we stay compatible with the entire NeuroML-software ecosystem: EDEN's simulation functionality is complemented by all the existing model-generation and results-analysis tools, and the ecosystem gets the most value out of EDEN as an interoperable simulator. Furthermore, positioning the simulator as a plug-compatible tool in the NeuroML stack allows us to focus our efforts on EDEN's features as a simulator (namely, computational performance, model generality, and usability). Finally, supporting an established modeling language makes user adoption much easier, compared to introducing a new simulator-specific language.

Another aspect that was taken into account in EDEN's design is *usability*. In addition to the benefits gained through NeuroML support, EDEN addresses usability through *automatic management* of multi-processing resources. This means that EDEN can distribute processing for a simulation across the processor cores of a personal computer–or even a computer cluster–fully automatically. Thus, users can fully exploit their modern computer hardware and deploy simulations of large networks on high-performance clusters, with no additional effort.

To evaluate all aforementioned features of EDEN, we employed: (1) qualitative benchmarks showing simulation fidelity to the standard neural simulator NEURON; and (2) quantitative benchmarks showing far superior simulation speed compared to NEURON, for networks of non-trivial size. The results of these benchmarks are expanded on in Section 3.

The contributions of this work are, thus, as follows:

- A novel neural simulator called EDEN supporting high model generality, computational performance, and usability by design.
- A novel model-analysis/code-generation technique that allows extracting the required calculations from a neural-network model, and casting them into efficient work items that can be run in parallel to simulate the network.
- A qualitative evaluation of EDEN, demonstrating NEURON-level fidelity, for a diverse set of neural models.
- A quantitative evaluation of EDEN, demonstrating simulation speeds of real-world neural networks (sourced from literature) up to close to two orders-of-magnitude faster than NEURON, when run on an affordable, 6-core desktop computer.

### 1.2. Qualitative Comparison of Neural Simulators

**Figure 1:**
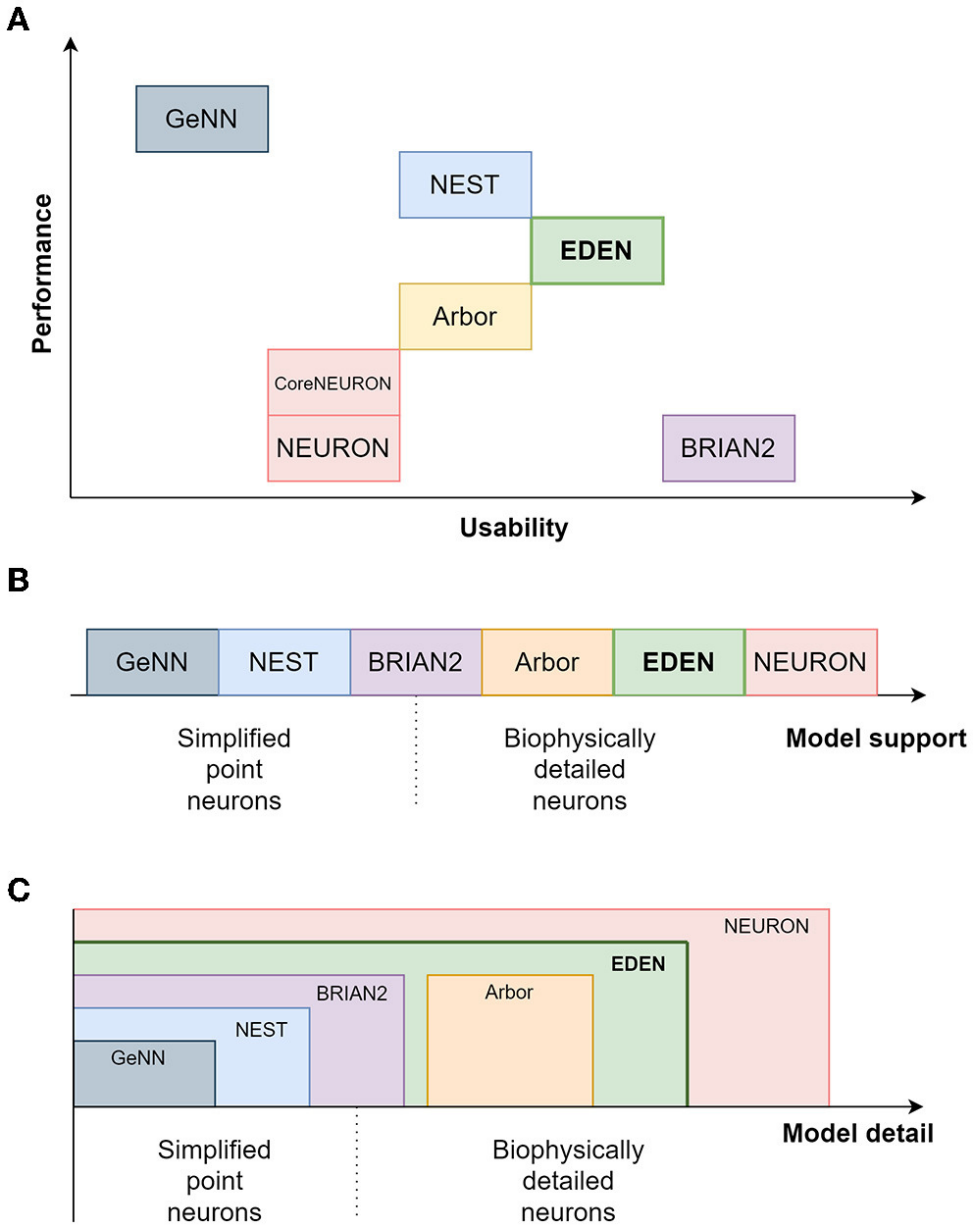
A relative comparison of the characteristics of EDEN and the established neural simulators. **(A)** compares the simulators on the performance and usability plane; **(B)** shows the ordering between simulators regarding the level of model detail; and **(C)** shows the level of modeling detail supported by each simulator.

In Table 1, we present a qualitative comparison between our proposed simulator EDEN, and the most popular, actively developed simulators in the computational-neuroscience field. In line with the scope of this article, we consider the more general-purpose simulators that can be used in a batch-mode, brain-modeling setting. The table consists of two parts, the top half dealing with coverage of neuron models and features, and the bottom half dealing with aspects of computational performance. Figure 1 also summarizes a qualitative comparison between the usability, range of supported models and computational performance of the various simulators. The characteristics and relative advantages of each simulator are further laid out in the following paragraphs.

**Table 1:** Qualitative comparison between EDEN and other state-of-the-art neural simulators: NEURON (McDougal et al., 2017), CoreNEURON (Kumbhar et al., 2019), JLEMS (Cannon et al., 2014), BRIAN2 (Stimberg et al., 2019), GeNN (Yavuz et al., 2016), NEST (Gewaltig and Diesmann, 2007), and Arbor (Akar et al., 2019).

	**EDEN**	**(Core) NEURON**	**Arbor**	**jLEMS**	**BRIAN2**	**NEST**	**GeNN**
**Supported models and features**							
LIF, AdEx, Izhikevich cells	✓	✓	Only LIF	✓	✓	✓	✓
Custom artificial cells	✓	✓	×	✓	✓	Partially *via* NestML	Partially *via* NineML
Highly detailed multi-compartmental cells	✓	✓	✓	×	Not practical	×	×
Native NeuroML support	✓	×	×	✓	×	×	×
Overall support compared to EDEN	Baseline	✓	×	×	×	×	×
**Performance**							
Machine-wide parallelism	✓	Manual	✓	×	Only for simple cases	✓	✓
Cluster-wide parallelism	✓	Manual	✓	×	×	✓	×
Cluster-wide auto-parallelization of detailed networks with graded synapses	✓	×	×	×	×	×	×
Overall performance compared to EDEN	Baseline	×	×	×	×	✓†	✓†

† *Only for artificial-cell models that NEST and GeNN support.*

NEURON (McDougal et al., 2017) is the popular standard simulator for biological and hybrid^2^ neural networks. It supports the richest set of model features among neural-simulation packages. A characteristic feature of NEURON is that everything about the model can be changed dynamically while the model is being simulated. This allows simulation of certain uncommon models, but it negatively impacts the simulator's computational efficiency. CoreNEURON (Kumbhar et al., 2019) is a new simulation kernel for NEURON that improves computational performance and memory usage at the cost of losing the ability to alter the model during simulation. It does not affect setting up the simulator and the model, which are still performed in the same way. Due to the underlying architectural design, the user has to add custom communication code to allow parallel simulation with NEURON, though there is ongoing effort to standardize and automate the needed user code (Dura-Bernal et al., 2019).

Compared to NEURON, EDEN only supports the NeuroML gamut of models. However, EDEN has much higher computational performance that also automatically scales up with available processor cores and computational nodes. Also, setting up a neural network in NEURON requires the connection logic to be programmed in its own scripting language. This is a cumbersome task and, what is more, NEURON's script interpreter is slow and non-parallel, often resulting in model setup taking more time than the actual simulation. In contrast, EDEN can load networks from any neural-network generation tool that can export to NeuroML^3^, thus leveraging the capabilities and computational performance of these tools.

Another simulator for biological neural networks is Arbor (Akar et al., 2019) which aims at high performance as well as model flexibility. Its architecture somewhat resembles the object model used by NEURON, which facilitates porting models, written in NEURON, to Arbor. However, compared to NEURON, it supports a smaller set of mechanisms. Regarding hybrid networks, modeling artificial cells is difficult; only linear integrate-and-fire (LIF) neurons are readily supported, and the user has to modify and rebuild the Arbor codebase for introducing new artificial-cell types. In addition, neuron populations connected by graded synapses cannot be distributed across machines for parallel simulation, which restricts scalability when running cutting-edge biological-neuron simulations. Compared to Arbor, EDEN supports about the same range of biophysical models but also supports *all types* of abstract-neuron models, while Arbor only supports LIF abstract neurons. This limitation prevents Arbor from supporting many hybrid networks. There is also a difference in usability: To set up a network model, the network-generation logic must be captured as Arbor-specific programming code. EDEN, instead, avoids simulator-specific programming by using a cross-tool file format.

In the space of artificial-cell-based spiking neural networks (SNNs), there are various specialized simulators in common use. jLEMS (Cannon et al., 2014) is the reference simulator for the LEMS side of NeuroML v2. It supports custom point-neuron dynamics through LEMS, which itself is a hierarchical-dynamics description language that co-evolved with NineML (Raikov et al., 2011). It was not designed for high performance and supports only simplified point neurons. BRIAN2 (Stimberg et al., 2019) is a simulator originally designed for point neurons, that focuses on usability and user productivity. It supports custom point-neuron dynamics, written in mathematical syntax. Its support for multi-compartmental cells is a work in progress; currently, all compartments must have the same set of equations. NEST (Gewaltig and Diesmann, 2007) and GeNN are general-purpose simulators for networks of point neurons and achieve high performance through a library of optimized codes for specific neuron types. Setting up the network is done through a custom programming language for NEST, and by extending the simulator with custom C++ code for GeNN. For NEST and GeNN, the way to add custom point-neuron types without modifying the C++ code is by writing the neuron's internal dynamics in a simulator-specific language; however, this method is not enough to capture all aspects of the model (such as multiple pre-synaptic points on the same neuron in NEST). Compared to abstract-cell simulators, EDEN has an advantage in model generality, since it also supports biophysically detailed multi-compartmental neurons, and hybrid networks of physiological and abstract cells. Although EDEN is not as computationally efficient as the high-end abstract-cell simulators, it readily supports *user-defined dynamics* inside the cells and synapses, whereas said high-performance simulators have to be modified to support new cell and synapse types. EDEN also supports non-aggregable synapses [i.e., not just types that can be aggregated into a single instance as per (Lytton, 1996)], and any combination of synapse types being present on any type of cell; which are also not supported by high-performance abstract cell simulators.

An important point to stress is that, the differences in supported model features, combined with the different model- description languages, make it difficult to reproduce the exact same neural network (and its output) across all simulators; this is especially the case for biologically detailed models. Thus, although there is much previous work on performance-driven neural simulation, our work is one of the first to directly compare performance with NEURON on physiological models that are drawn from existing literature, rather than employing synthetic ones. This further underscores the point that EDEN is a general-purpose tool that can be readily used with existing NeuroML models as well as in new NeuroML projects.

In the literature, the designers of CoreNEURON and Arbor have each reported utilizing the cores of a whole High-Performance Computing (HPC) node, to achieve up to an order of magnitude of speedup over NEURON. While the models and the machines used in those cases are not identical to ours for allowing a strict comparison, our demonstrated speedup of 1 to nearly 2 orders of magnitude over NEURON on a 6-core PC shows that EDEN is more than competitive against the state of the art in terms of computational performance. Furthermore, the fact that a regular desktop PC has been used for achieving such speedups makes the results highly relevant for a typical neuroscientist's computational resources.

## 2. Methods and Materials

### 2.1. EDEN Overview

**Figure 2:**
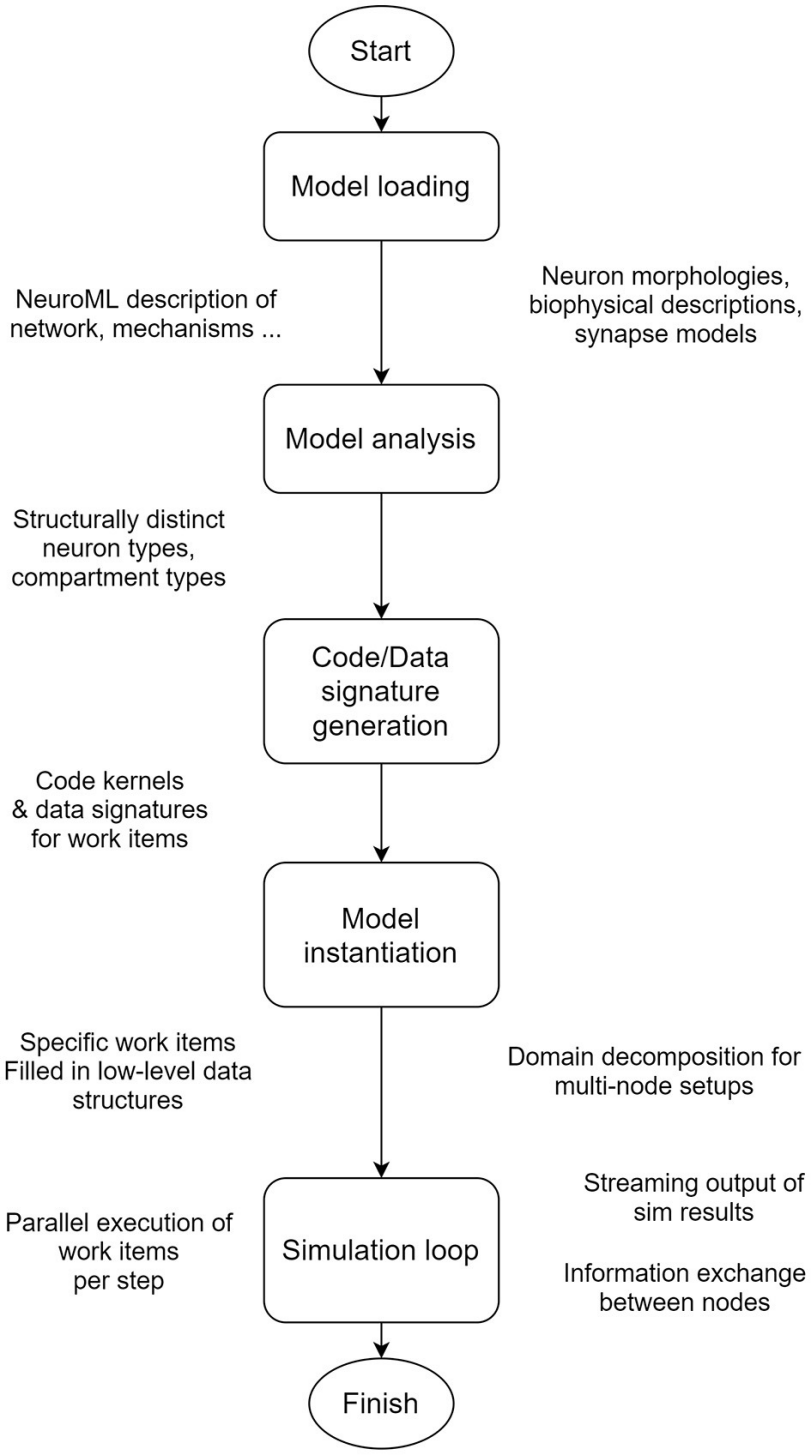
EDEN's processing pipeline. The whole model is analyzed in order to extract the computationally similar parts of neurons, and to generate optimized code and data representations for them, on the fly.

The architecture of EDEN can be visualized as a processing pipeline, which is illustrated in Figure 2. The pipeline details and the reasons for allowing EDEN to deliver high performance, model flexibility, and usability are explained in this section. While current neural simulators primarily focus on either computational performance or model generality, EDEN simultaneously achieves both objectives with a novel approach: it generates efficient code kernels that are tailored for the neuron models at hand.

EDEN performs time-driven simulation of any sort of neural network that can be described in NeuroML. To enable simulation of complex, and often heterogeneous, networks with high performance, EDEN first performs model- and workload-analysis steps so as to divide the simulation workload into independent, parallelizable components and, subsequently, determines *efficient code and data representations* for simulating each one of them. Finally, EDEN employs automatic code generation to convert these components to parallel-executable tasks (called work items). Code generation boosts computational efficiency by adapting performance-critical code to the specific model being simulated and to the specific hardware platform being used. Task parallelization boosts computational efficiency even further by distributing the simulation work across multiple CPU cores in a given computer, and across multiple computers in a high-performance cluster.

### 2.2. Usability Through Native NeuroML Support

Choosing NeuroML as EDEN's input format allows us to focus on our core part of high-performance numerical simulation and, at the same time, leverage the existing NeuroML-compatible, third-party tools for design, visualization, and analysis of neural networks. Adopting the standard also improves the simulator's usability, as the end user does not need to learn one more simulator-specific modeling language. In practice, directly supporting the NeuroML standard also allowed us to verify the simulator's results against the standard NEURON simulator, for numerous available models. As we will see in Section 3, the same NeuroML description can be used for both simulators and run automatically. Otherwise, porting all these models separately to both simulators would have taken an impractical amount of effort, making verification and comparison much more difficult to achieve.

### 2.3. Performance and Flexibility Through Code Generation

Neural models, especially biophysical ones, are commonly described through a comprehensive, complex hierarchy of mechanisms. Neural-simulation programmers have to consider this cornucopia of mechanisms and their combinations so as to form neural models. All the while, the formulations behind the mechanisms are constantly evolving, thus, allowing for no single set of mathematical equations to cover most (or even a few of the) neural models.

The resulting complexity—in both setting up a model and running the simulation algorithm—has steered general-purpose neural-simulation engines to adopt *object-oriented models* of the neural networks being run. Each type of programming object, then, captures a respective physiological mechanism, and the hierarchy of mechanisms in the model is represented by an equivalent object hierarchy. By adopting NeuroML, EDEN takes the same object-oriented approach at the model input.

Although this approach does help simplify the *programming model* by mitigating the conceptual and programming complexity of working with sophisticated models, it is detrimental to the *execution model* since it is an inefficient way to run the simulations on modern computer hardware. The object-oriented data structure of a model in use has to be traversed, every time the equations of the model are evaluated and the model's state is advanced. The traversal logic in use enforces a certain ordering among the calculations that are needed to advance the model's state. Also, the object-oriented model's pointer-based data structures make control flow and data-access patterns unpredictable, slowing down the processing and memory subsystems of the computer, respectively.

For example, NEURON advances the state of the network in successive stages: Within the scope of one parallel thread, the processing stages of (a) evaluating current and conductivity for all membrane mechanisms present, (b) solving the cable equation for all neurons, and (c) advancing the internal state of all membrane mechanisms are performed in strict sequence. Since each part of these three stages pertains to a specific compartment of the network, and yet processing of these stages for the same compartment is separated in time by processing for the whole network, this ordering is detrimental to data locality. In accelerator-enabled implementations of this technique, namely CoreNEURON and Arbor, the mechanisms with identical mathematical structure are grouped together and executed in an even stricter sequence within the original phases of processing. This exacerbates the impact to locality and introduces synchronization overhead that increases with model complexity, as parallelization is only applied across identical instances of each mechanism type.

Now, starting from the computer-architecture part of the problem, HPC resources are designed so that the maximum amount of computations can be done independently and simultaneously. Thus, fully utilizing them requires streamlined algorithms and *flat data structures*. In many cases, neural-simulation codes have been custom-tailored for the HPC hardware at hand. Although such codes improve simulation speed and supported network size by orders of magnitude compared to general-purpose simulators, they make inherent model assumptions that *prevent* them from supporting other models. The result is that these manually optimized codes, as well as the knowledge behind them, are abandoned after the specific experiment they were developed for is concluded.

To avoid the pitfalls of these two approaches, EDEN consciously refrains from imposing a specific execution model, so that it can support both model generality and high-performance characteristics. Both of them are simultaneously achieved through a novel approach: efficient code kernels that are tailored for the neuron models at hand are automatically generated, while supporting the whole NeuroML gamut of network models. The specific processing stages that EDEN undergoes to achieve this (see Figure 2) are as follows:

1. Analyse all types of neurons in a given model.
2. Deduce the parts of the neural network that have a similar mathematical structure.
3. Produce efficient code kernels, each custom-made to simulate a different part of the network.
4. Iteratively run the code kernels to simulate the network.

This code-generation approach used by EDEN has manifold benefits: First, the simulation can be performed without traversing the model's hierarchy of mechanisms at run time, since the set of required calculations has already been determined at setup time. Second, since the generated code contains only the necessary calculations to simulate a whole compartment or neuron, the compiler is given much more room for code optimization compared to code generation for individual mechanisms. Third, the minimal set of constraints that EDEN's backend places on the code of work items allows incorporating hand-written code kernels that have been optimized for specific neural models. This is also made possible due to the model-analysis stage, which isolates groups of neurons and/or compartments with an identical mathematical structure; when a hard-coded kernel is available for a detected neuron type, it can be employed for the specific cell population, to further boost performance. Thus, EDEN's model-decomposition and code-generation architecture delivers high computational performance for a general class of user-provided neuron models, and it also permits *extensions* in both the direction of model generality and computational performance.

For this first version of EDEN, a *polymorphic kernel generator*^4^ that supports the full gamut of NeuroML models was implemented. The specifics of the code kernels are customized for each neuron type; still, the generator's format covers any type of neuron, whether it is a rate-based model, an integrate-and-fire neuron or a complex biological neuron, or whether the interaction is event-based, graded, or mixed. Thus, this implementation provides a baseline of computational efficiency, for all neural models. It can also work in tandem with specialized kernels. Two ways of extending EDEN with such specialized high-performance codes are described below.

The simplest way to integrate an existing code in EDEN is to directly use it just for the models that the code supports. Programming-wise, the neural network to be run is checked whether it can be run on the new code, and if this is the case, the original new code is generated as a work item, and the simulation data is accordingly allocated and initialized for the model. By running the same code on the same data, extended EDEN should perform as well as the original EDEN code, for the supported family of models.

Alternatively, if the specialized code applies to only a part of the desired network, it can interface with work items from EDEN's general-purpose implementation (or other extensions) for the part of the network that it does not cover. Some modification is then necessary to make the code exchange information (such as synaptic communication) in the same way as the work items it is connected to, but the gains in model generality are immediate.

Following these methods, the usefulness of the optimized code is extended with the least possible effort, simulation can utilize multiple computational techniques at the same time, and the details of each technique do not affect the rest of the EDEN codebase.

### 2.4. EDEN Concepts

#### 2.4.1. Work Items

The fundamental units of work executed per each simulation step in EDEN are called “work items.” The work items are parts of the model that can be processed in parallel within a simulation step, to advance the state of the simulated model. Within a time-step, each work item is responsible for updating a small part of the entire model. Each work item is associated with a single part of the model data being simulated, and a single code block being run. That code block is responsible for updating the mutable part of its model data over time, but it may also update other parts as well, so that it can send information to other parts of the model. One such case is transmission of spike events to post-synaptic components. Then, data-access collision with the work item that is assigned to the post-synaptic component is avoided by double buffering; the work item receiving the information reads it on the next time-step, while leaving the alternate buffer available for other work items to write to. In the case multiple other work items may write simultaneously, atomic memory accesses are used.

In this first version of EDEN, each work item involves simulating exactly one neuron, but the design allows further variations—for example, to consolidate simple neurons in batches, or to split large neurons in parts—as long as the calculations for each work item are independent.

#### 2.4.2. Code and Data Signatures

EDEN generates compact code and data representations to run the simulation, by composition of the multiple underlying parts. The details of how this works are explained below.

Each simulated mechanism is defined by its dynamics, the fixed parameters and state variables of the dynamics, and the variables through which it influences other mechanisms, and is influenced by other mechanisms. The external variables influencing the mechanism are called *requirements*, and the values it, in turn, presents for other mechanisms to use, are called *exposures*. Then, to simulate the mechanism, the required actions are:

- to evaluate all variables involved in the dynamical equations (called “assigned” henceforth, in EDEN as well as NEURON parlance). This is the “evaluation” step of the simulation code.
- then, to advance the simulation's state based on the dynamics, and the current values of the assigned variables. This is the “update” step of the simulation code.

**Figure 3:**
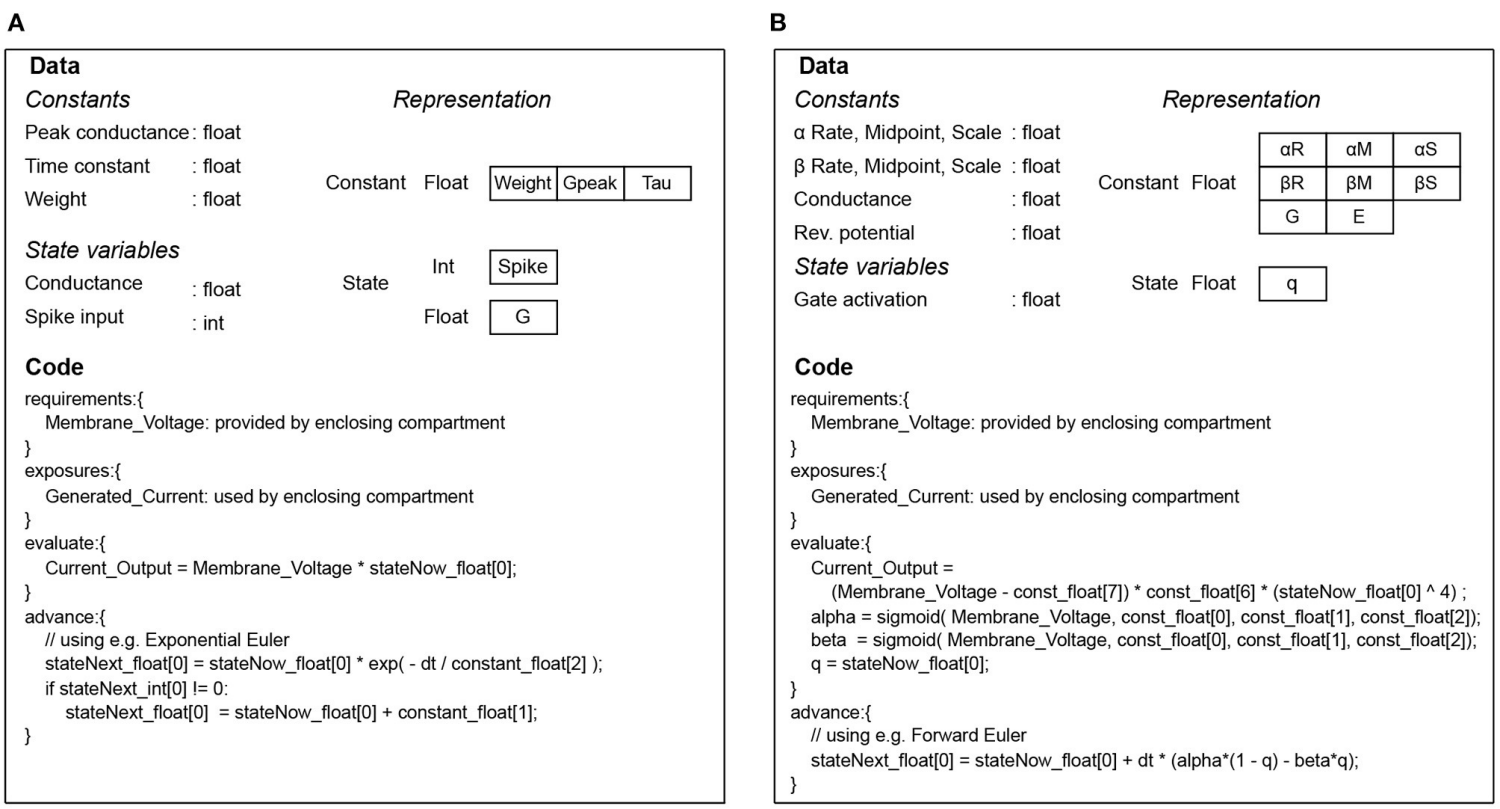
Code and data signatures for an exponential-conductance post-synaptic component **(A)**, and for a classic Hodgkin-Huxley sodium channel **(B)**.

The whole set of code and data for simulating a mechanism is collectively called a *signature* in EDEN parlance. Examples of code-data signatures are shown in Figure 3, for simple cases of a post-synaptic component and an ion channel. Each of them consists of the code for running the “evaluation” and “update” steps (also called *code signature*), and the data representing the mechanism (also called *data signature*). The signature representation is used in EDEN both for simple mechanisms and composite ones. In fact, the signatures of smaller mechanisms are successively merged to form the signatures of higher-order parts of the neurons, eventually forming signatures for whole compartments or even entire neurons. The code of such signatures is then run in parallel, in order to simulate the whole neural network.

In order to combine the signatures representing two mechanisms, the interfaces (i.e., requirements and exposures) through which the mechanisms interact have to be determined. The hierarchical structure of the provided neural models helps in this, since it delineates the interfaces through which the “parent” mechanism interacts with its “children,” and the “siblings” interact with each other.

Code generation starts from the simple, closed-form mechanisms present (for example, Hodgkin-Huxley rate functions or plasticity factors of mechanisms). The hierarchy of mechanisms present in a neuron is traversed, and signatures are incrementally formed for each level of the hierarchy.

The specific steps to merge two signatures are then, in terms of code and data:

- The “evaluation” parts of the code signature have to be placed in a certain order, such that after the variables each mechanism requires are defined and evaluated before the mechanism's evaluation code.
- The “update” parts of the code signature can be appended anywhere after the mechanism's corresponding evaluation code.
- The data signatures of the mechanisms are simply concatenated to each other.

As signatures are generated for each higher or lower level mechanism, an auxiliary data structure that has the same hierarchical structure as the original object-oriented model is also formed. This is called the *implementation* of the signature, and it keeps track of how the conversion to signatures was performed, for each mechanism. Relevant information includes the specific decisions made for the generated code (like selection of ODE integrator for the particular mechanism), and the mapping of abstract parameters and state variables (such as the gate variable of an ion channel, the fixed time constant of a synapse, the membrane capacitance of a compartment, etc.) to the specific variables allocated in the data signature. The information is useful for:

- referring to parts of the network symbolically (like when recording trajectories of state variables, and when communicating data-dependencies between machines in multi-node setups),
- initializing the data structures through the symbolic specifications in use (such as weights of specified synapses),
- properly combining signatures, according to implementation decisions (e.g., adjusting the update code to the integrator in use).

#### 2.4.3. Data Tables and Table-Offset Referencing

To achieve high performance during simulation, EDEN uses a simplified data structure for the model. The model's data are structured in a set of one-dimensional arrays of numbers. These arrays (called *tables* henceforth) are grouped by numerical type (such as integral or floating-point), and mutability (whether their values remain fixed along the simulation, or they evolve through time). This means that each value in the model being simulated is identified by the table it belongs to, its position in the table, and the value's numerical type and mutability.

The value's location can then be encoded into an integer, from the table's serial number and the offset on the table. The code generated by EDEN can use such references to values at run time, to access data associated with other work items. This relieves EDEN's simulation engine from the need to manage communication between parts of the model with a fixed implementation. Instead, control is given to the work items' generated code on how to manage this communication effectively.

Another benefit of the table-offset referencing scheme is that the references can be redirected to any location in the model's data, if need arises. This is used in particular when a model is run on a computer cluster, where parts of the network are split between computers. In this case, only a fraction of the model is realized on each machine, and the data read by or written to remote parts of the network are redirected to local mirror buffers instead. The change is automatically applied by editing the references in the instantiated data, hence there is no need to change the generated code for the work items.

### 2.5. Implementation

The present implementation of EDEN takes as input NeuroML and supports all neural models in the NeuroML v2 specification. This implementation, and the code kernels it generates, can be used as a fall-back alternative to further extensions: the extensions can provide specialized implementations for specific parts of the neural network, while the rest of the network is still covered by the fully general, original implementation.

#### 2.5.1. Structure of the Program

To begin analysis and simulation of a neural network, its NeuroML representation, along with additional LEMS components describing the custom neuron mechanisms present, is loaded into an object-oriented representation.

The main steps of the process are:

1. Model analysis
2. Work-item generation through code and data signatures
3. Model simulation in the EDEN simulation engine

These steps are further described in the following sections.

#### 2.5.2. Model Analysis and Code Generation

The first part in model analysis is to associate the types of synapses in the network with the neuron types they are present in. This resolves which types of neurons contain which kinds of synaptic components, and where each kind of synapse is located on the neuron. The same assessment is also made for the input probes connected to each neuron, since probes are also part of the neurons' models.

Then, each neuron type is analyzed, to create a signature for each kind of neuron. First, the structure of the neuron is split into compartments, and the biophysical mechanisms applied over abstract groups of neurite segments are made explicit against the set of compartments. Thus, for each compartment, we get the entire list of biophysical mechanisms existing on it. Using these lists, the corresponding code and data signature is formed for each individual compartment.

**Figure 4:**
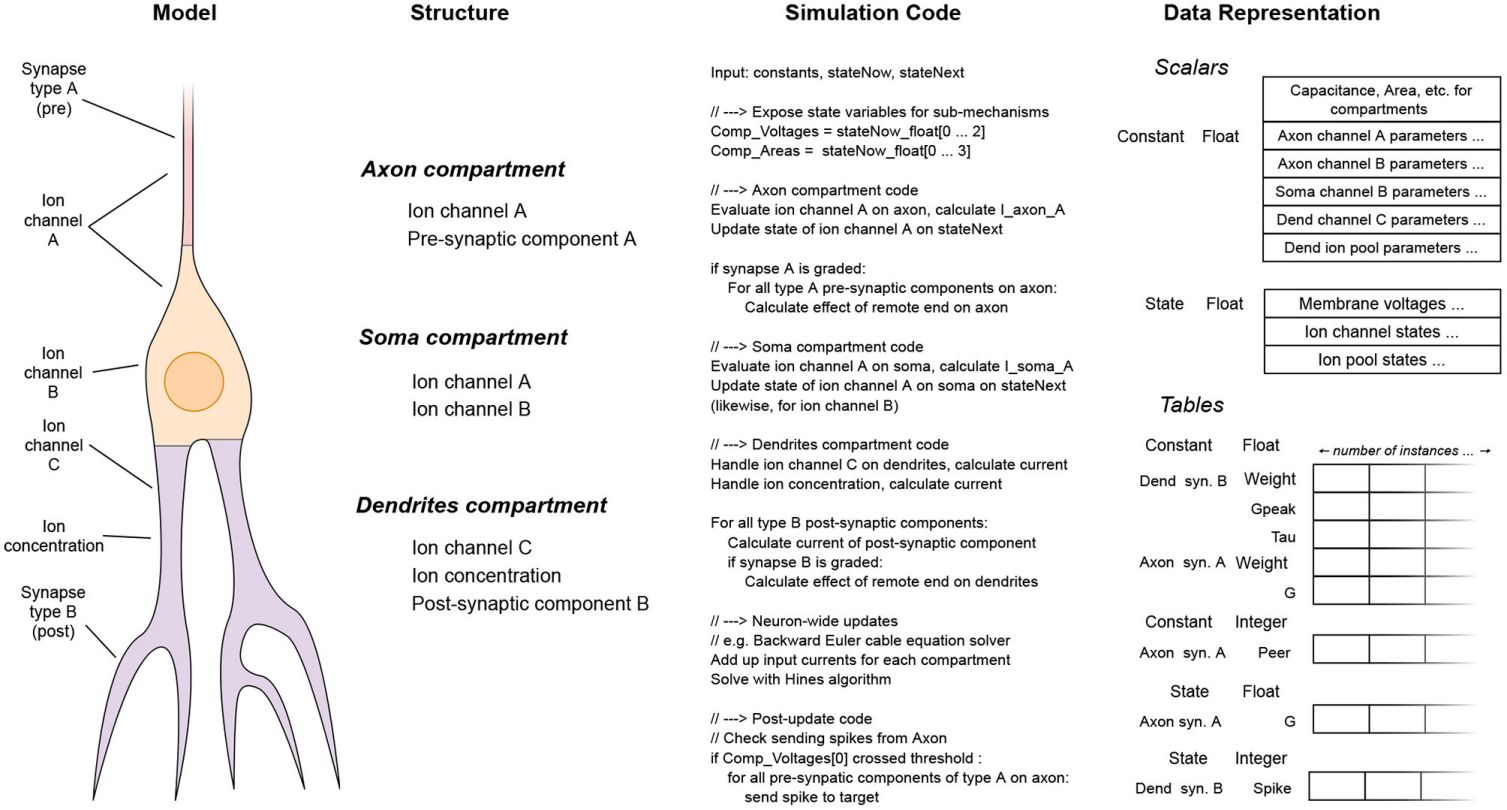
The stages of the per-neuron signature synthesis process, for neurons with few (phenomenological) compartments. The neuron shown consists of three different compartments, each containing different physiological mechanisms. The simulation code for all mechanisms is laid out in a flat format, along with their associated data. Thus, a streamlined and compact code kernel is created for this specific type of neuron.

If the number of compartments is small, these signatures are concatenated for all compartments present on the neuron, into a neuron-wide signature. This way, a compact code block is generated, with a form similar to how hand-made codes are written for reduced compartmental neuron models. The process is illustrated in Figure 4.

##### 2.5.2.1. Signature Deduplication for Identical Compartments

**Figure 5:**
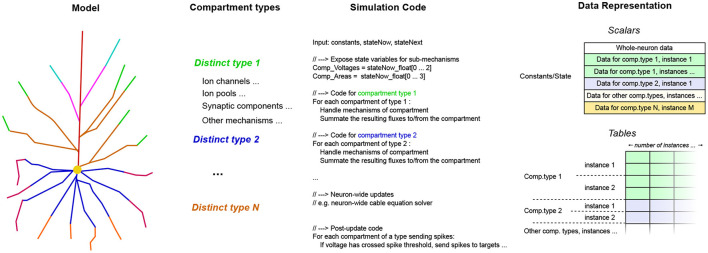
The stages of the per-compartment signature de-duplication process, from the abstract model to the concrete implementation. On the schematic of the detailed neuron model, distinct compartment types are shown in different colors. The components sharing the same type are then grouped together, in terms of simulation code and data representation. The specific mechanisms comprising the compartments and the data cells they contain are not shown here, for brevity.

If the number of compartments is large, it is not practical to generate a flat sequence of code instructions for each individual compartment. However, in practice, neuron models have less than a few tens of distinct compartment types with different mathematical structure, in the most complicated models. Thus, a different approach called signature de-duplication is employed, as follows. In this approach, the compartments are grouped for processing, according to their structural similarity (equivalently, similarity of signature representation). The process is illustrated in Figure 5.

Using the per-compartment list of mechanisms, we can immediately deduce which compartments have the exact same structure; which is the case when the set of mechanisms, and thus the code and data signature representation, is the same. The code signature for the whole neuron now has a set of loops, one for each type of compartment. Inside the loop, the code signature to simulate a single compartment is expanded. Each iteration of the loop performs the work for a different compartment with the same structure. Thus, the data signatures are concatenated together for each group of compartments, and the appropriate offsets are shifted in each iteration of the loop, so that they point to the specific instance of the per-compartment data signature to be used each time. By generating a specific code block for each sort of compartment, we eliminate the computational overhead of traversing the individual mechanisms present on each simulation step, that affects previous general-purpose neural simulators. Finally, after the code signatures for the work items are determined, they are compiled to machine code, and loaded dynamically on the running process.

#### 2.5.3. Model Instantiation

**Figure 6:**
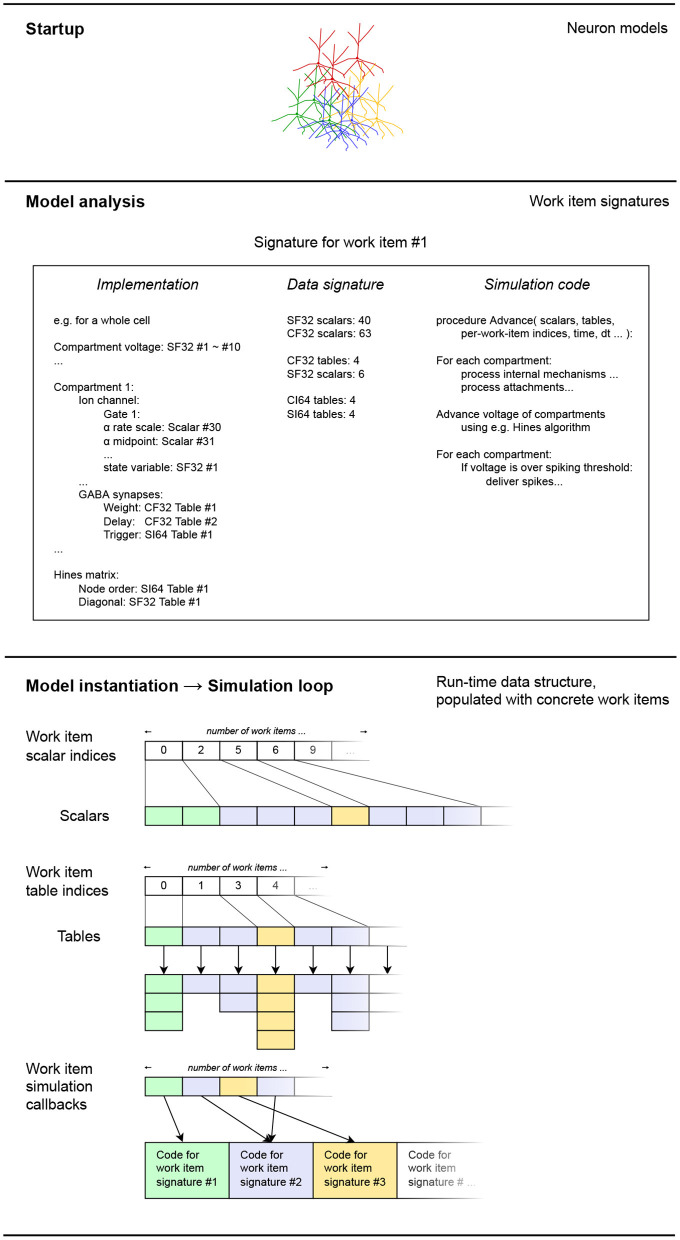
A schematic representation of how the extracted work item signatures are converted to low-level data structures for efficient processing. For each work item, the set of scalars and work tables of each is appended into flat node-wide arrays, for each data type. Data types shown: CF32 = 32-bit floating-point constants, CF64 = 64-bit integer constants, SI64 = 64-bit integer variables. The different data types for scalars and tables have been omitted for clarity in the diagram, without loss of generality. Colors indicate different code-data signatures among work items.

After the model is analyzed to determine the structure of the work items it is converted to, it is time for the work items and their associated data to be realized in memory. The process that we describe in the following is also illustrated on Figure 6. As mentioned previously, in this version of EDEN, each neuron in the network, along with the synaptic components and input probes attached to it, is assigned to an individual work item. The mapping of parts of the network to work items, is thus fixed.

The data signatures of the work items specify the number of scalar variables and tables each work item uses. Thus, to instantiate each work item, we just have to allocate the same number of scalars and tables, and keep track of the work item for which these blocks of memory were allocated. After the variables are allocated for each work item instance, they are filled in, according to the model definition. This is made possible by the implementations of the work items, that keep track of how model-specific references to values map to concrete data values for each work item. Thus, the changes between different instances in the specified model are mapped into changes in the low-level data representation.

The scalar values for each instantiated work item are located in contiguous slices of certain tables, which are reserved for each type of scalar values. Other parts of a work item may not have a fixed size every time. This is, for example, the case for synaptic components of a given type; they may exist in multitudes on a compartment of a neuron, and their number varies across instances of the neuron or compartment type. The data for these variable-sized populations is stored in tables; one set of tables per kind of mechanism on the same compartment. That way, although the sizes of each set of tables may vary eventually, the number of scalars and individual tables required for a work item remains fixed, for all of its instances.

After allocating the scalars and the tables for the model, what remains is to replace default scalar values with per-instance overrides specified by the model where they exist, and to fill in the allocated tables with their variable-sized contents. The customized scalar values and tables pertaining to the inner models of neurons (where the “inner model” excludes the synapses and input probes attached to the neuron) are filled in while running through the list of neurons specified in the model. The synaptic connections in the network model are also run through, and the corresponding pre- and post-synaptic components are instantiated on the connected cells. More specifically, on each cell, the tables representing the specified synaptic component are extended by one entry each, with the new entries having the values of the scalar properties of the mechanism. The default values for these properties are provided by the data signature of the mechanism, and customized values (such as weight and delay of the synapse) are filled in using the connection list in the model description.

#### 2.5.4. Simulation Loop

After model instantiation is done, the code blocks and data structures for the model are set up in system memory and ready to run. Communication throughout the network is internally managed by the code blocks, *via* a shared-memory model. Double buffering is employed to allow parallel updates of the state variables within a time-step, thus all state-variable tables are duplicated to hold the state of the both the old and new time-step as the latter is being calculated.

All that remains to run the simulation, is to repeat the following steps for each simulation time-step:

- set the global “current time” variable to reflect the new step;
- execute the code for each work item in parallel, on the CPU;
- output the state variables to be recorded in the network, for the new time-step;
- alternate which set of state variable buffers is read from and written to, as per the common double-buffering scheme.

Parallel execution of the code kernels is managed by the OpenMP multi-threading library. The “dynamic” load-balancing strategy is followed by default, so whenever a CPU thread finishes executing a work item, it picks the next pending one. The synchronization overhead of this load-balancing strategy is mitigated by the relatively large computational effort to simulate physiological models of neurons, as will be shown in Section 3.

#### 2.5.5. Numerical Methods

The numerical integration methods that EDEN employs in this version are simple but they are sufficient to provide accurate simulation, as we will demonstrate in Section 3. All calculations are done with single-precision arithmetic except for expressions involving the amount of simulated time, which is represented with double precision since it changes by microseconds throughout up to hours. The state of synapses and of most membrane mechanisms is advanced using the Forward Euler integrator. An exception is made for the gate variables of Hodgkin-Huxley ion channels with alpha-beta rate (or, equivalently, tau-steady state) dynamics, where NEURON's cnexp integrator (i.e., Exponential Euler under the assumption that the transition rates are fixed throughout the timestep) is employed. To simulate the diffusion of electrical charge within each cell, we use a linear-time, Gaussian-elimination method that is equivalent to the Hines algorithm (Hines, 1984) used in NEURON.

#### 2.5.6. Running on Multi-Node Clusters

Apart from the high-performance properties implemented in EDEN for fast simulation on a single computer, EDEN also supports MPI-based execution on a compute cluster, so as to further handle the large computational and memory needs of large simulations. To distribute the simulation over multiple co-operating computational nodes, some modifications are made to the process described above. In the following, each co-operating instance of EDEN is called a “node.”

At the model-instantiation stage, the nodes determine which one will be responsible for simulating each part of the neural network. The neurons in the network are enumerated, and distributed evenly among nodes. To keep a small and scalable memory footprint, in this version of EDEN, each node is responsible for a contiguous range of the enumerated sequence of neurons. Then, each node instantiates only the neurons it is responsible to simulate, allocating the corresponding scalar values and tables. The parts that pertain only to individual neurons are also instantiated and filled in. But special care has to be taken when instantiating synapses, since they are the way neurons communicate with each other—and the neurons a node is managing may communicate with other neurons, that are managed by a different node. Thus, the instantiation of synapses is performed in three stages:

1. an initial scan of the list of synapses, to determine which information is needed by each node from each node during the simulation;
2. exchange of requirement lists among nodes, so they all are aware of which pieces of information they must send to other nodes, during the simulation;
3. establishment of cross-node mirror buffers, and remapping cross-node synapses so that they use these buffers, to access the non-local neurons they involve.

To support these stages, a new auxiliary data structure is created on each node. It is an associative array, mapping the identifiers of peer nodes to the set of information that needs to be provided by that node to run the local part of the simulation (called *send list* from now on). A send list consists of the spike event sources and state variables on specific locations on neurons, that the node needs to be informed about to run its part of the simulation. The kinds and locations for these state variables and spikes, are stored and transmitted using a symbolic representation, that is based on the original model description. For example, a location on a neuron is represented by the neuron's population and instance identifiers, the neurite's segment identifier, and the distance along that segment from the proximal to the distal part. Using symbolic representations for send lists allows each node to use the most efficient internal data representation for its part of the model, without requiring peer nodes to be aware of the specific data representation being used on each node. The three stages to set up multi-node coordination are further described in the following:

##### 2.5.6.1. Synapse-Instantiation Stage

First, the list of synaptic connections is scanned, and synapses connecting pairs of neurons are handled by each node according to four different cases:

1. If a synapse connects two neurons managed on this node, it is instantiated, and the tables are filled in just as described above, for the single-node case.
2. If neither neuron connected by the synapse is managed by this node, the synaptic connection is skipped.
3. If the local neuron needs to receive information from the remote neuron (as is the case with post-synaptic neurons and those with bi-directional synapses), then the location on the remote neuron and type of data (e.g., spike event or membrane voltage), is added to the send list for the remote node. The local neuron's synaptic mechanism is also instantiated using its data signature, however:
  - If the synaptic mechanism is continuously tracking a remote state variable (as is the case with graded synapses), the table-offset reference to that variable is set with a temporary dummy value. This entry is also tracked, to be resolved in the final synapse fix-up stage.
  - If the mechanism receives a spiking event from a remote source (as is the case with post-synaptic mechanisms), the mechanism receives the spike event in one of its own state variables, instead of tracking a remote variable. (This is the same way event-driven synapses are implemented in the single-node case.) The state variable is used as a flag, so custom event-based dynamics are handled internally. Thus, this entry has to be tracked, so that its flag can be set whenever the remote spike source sends a spiking event, at runtime.
4. If the locally managed neuron does not need to receive information, then is it skipped. The need for this node to send information to other peers will be resolved in the following send-list exchange stage.

##### 2.5.6.2. Send-List Exchange Stage

At this point, the send lists have been determined, according to the information each node needs from the other nodes. These send lists are then sent to the nodes the data is needed from; sending nodes do not have to know what they are required to send *a priori*. Therefore, the algorithm described in the following also applies to the more general problem of distributed sparse multigraph transposition (Magalhães and Schürmann, 2020).

In the beginning of this stage, each node sends requests to the nodes it needs data from; each request contains the corresponding send list it has gathered. Then, from each node it sent a request to, it awaits an acknowledgement. While nodes are exchanging send lists, they also participate asynchronously in a poll of whether they have received acknowledgements for all the requests they sent. When all nodes have received all acknowledgements, this means all send lists have been exchanged, and the nodes can proceed to the next stage.

By using this scheme, information is transmitted efficiently in large clusters: no information has to be exchanged between nodes that do not communicate with each other. This is a scalability improvement over existing methods, where the full matrix of connectivity degrees among nodes is gathered on all nodes (Vlag et al., 2019; Magalhães and Schürmann, 2020).

##### 2.5.6.3. Synapse Fix-Up Stage

After all data dependencies between nodes are accounted for, each node allocates communication buffers to send and receive spike and state-variable information. The buffers to receive the required information are allocated as additional tables in the data structures of the model. They are “mirror buffers” that allow each node to peek into the remote parts of the network they need to.

The table-offset references that were left unresolved in the synapse-instantiation stage because the required information was remote, are now updated with references to the mirror buffers for the corresponding remote nodes. This way, the components of cross-node synapses that—were the simulation run on a single node—would directly access the state of adjacent neurons, now access these mirror buffers instead. The mirror buffers are, in turn, updated on every simulation step as described in the next section, maintaining model integrity across the node cluster.

##### 2.5.6.4. Communication at Run-Time

After the additional steps to instantiate the network on a multi-node setup, the nodes also have to communicate continuously during the simulation. Each node has to have an up-to-date picture of the rest of the network its neurons are attached to, to properly advance its own part of the simulation. Thus, the simulation loop is extended with two additional steps: to send local data to other nodes that need them, and to receive all information from other nodes it needs to proceed with the present time step.

The nodes follow a peer-to-peer communications protocol, which resembles the MUSIC specification (Ekeberg and Djurfeldt, 2008). The data sent from each node to a peer per time-step form a single message, consisting of:

- A fixed-size part, containing the values of state variables the receiving node needs to observe.
- A variable-size part, containing the spike events that occurred within this communication period. The contents are the indices of the events that just fired, out of the full list of events previously declared in the send list.

During transmission, each data message is preceded by a small header message containing the size of the arriving message; this is done so that the receiving node can adjust its message buffer accordingly.

After receiving the data message, the fixed-size part is directly copied to the corresponding mirror buffer for state variables, while the firing events in the variable-sized list are broadcast to the table entries that receive them. Broadcasting of firing events is performed using the spike recipient data structure that was created in the synapse instantiation stage.

Inter-node communications are placed in the simulation loop, as follows:

- In the beginning of the time step, the information to be sent to other nodes is picked from this node's data structures, into a packed message for each receiver. Transmission of these packed messages begins;
- Meanwhile, the node starts receiving the messages sent by other nodes to this one. Whenever a message arrives, it is unpacked and the contents are sent to mirror buffers and spike recipients in the model's data.
- When messages from all peers for this node are received, the node can start running the simulation code for all work items, while its own messages are possibly still being sent;
- Before proceeding with the next simulation step, the node waits until all messages it started sending have been fully sent; so, then, the storage for these messages can be re-used to send the next batch of messages.

## 3. Results

During development of the EDEN simulator, we ran functional and computational performance tests, using NeuroML models from the existing literature. The functional tests were used to ensure that the simulator properly supports the various model features specified by NeuroML, and that its numerical techniques are good enough, with regard to stability and numerical accuracy.

The NeuroML-based simulations used in the experiments here were sourced from the Open Source Brain model repository (OSB) (Gleeson et al., 2019), and from the NeuroML-DB (Birgiolas et al., 2021). They were selected to cover a wide range of models in common use (regarding both level of detail and model size), and because their results clearly show various features of neural activity, and how each simulator handles them.

The simulations for the functional tests included all neuron models available on the NeuroML-DB and also present in the more general ModelDB (McDougal et al., 2017), and the smaller version of each network used in the performance tests. A visual comparison of output trajectories for various other OSB models is included in the Supplementary Material, in order to illustrate some finer details of the differences between the simulators. The performance tests were done on large neural networks in order to evaluate EDEN's computational efficiency and scalability, in various realistic cases.

Both simulation accuracy and performance characteristics were compared to the standard NeuroML simulation stack for biophysical models: the NEURON simulator (version 7.7), with the NeuroML-to-NEURON exporter jNeuroML (version 0.10.0). Model-porting complications were thus avoided by using the same NeuroML model descriptions. NEURON is the most commonly used general-purpose neural simulator, its numerical algorithms have been proven through decades of use, and it also enjoys the most complete NeuroML support to date (through the jNeuroML tool).

### 3.1. Evaluation of Functional Correctness

#### 3.1.1. Evaluation Through Single-Neuron Models

Each neuron model present in the NeuroML-DB and also present in the ModelDB was individually simulated, to compare EDEN's results with NEURON's in each case. The protocol used was to stimulate each neuron with a 2 nA DC current clamp on its soma, from 10 to 90 ms of simulated time, with total simulation time being 100 ms. A fixed timestep of 0.025 ms was used for all simulations. The one recorded waveform for each neuron was membrane voltage on the soma. This is one type in the array of tests already used in the NeuroML-DB, to characterize the electrophysiology of each neuron model.

The similarity metrics being assessed for EDEN's resulting waveforms, using NEURON's waveforms as reference, are:

- per-cent difference in inter-spike interval (ISI), assuming a spike threshold of −20*mV*,
- the NeuroML-DB similarity metric $1-\frac{mean\left(\left|x-\hat{x}\right|\right)}{max\left(x\right)-min\left(x\right)}$, where *x*, $\hat{x}$ are the reference and tested waveforms. This one is used throughout NeuroML-DB to measure the discrepancy of NEURON's results under different (fixed) simulation step sizes, to determine an optimal step size that balances error with simulation time.

In total, EDEN failed to run seven models, whereas jNeuroML failed to run 24 models, out of 1,105 neuron models in total. EDEN could not run said seven models because they contain minor LEMS features it does not support at the time—though all these models can still work with a minimal, equivalent change to their description. We speculate that jNeuroML could not run said 24 models because of a defect in its support for certain types of artificial cells.

**Figure 7:**
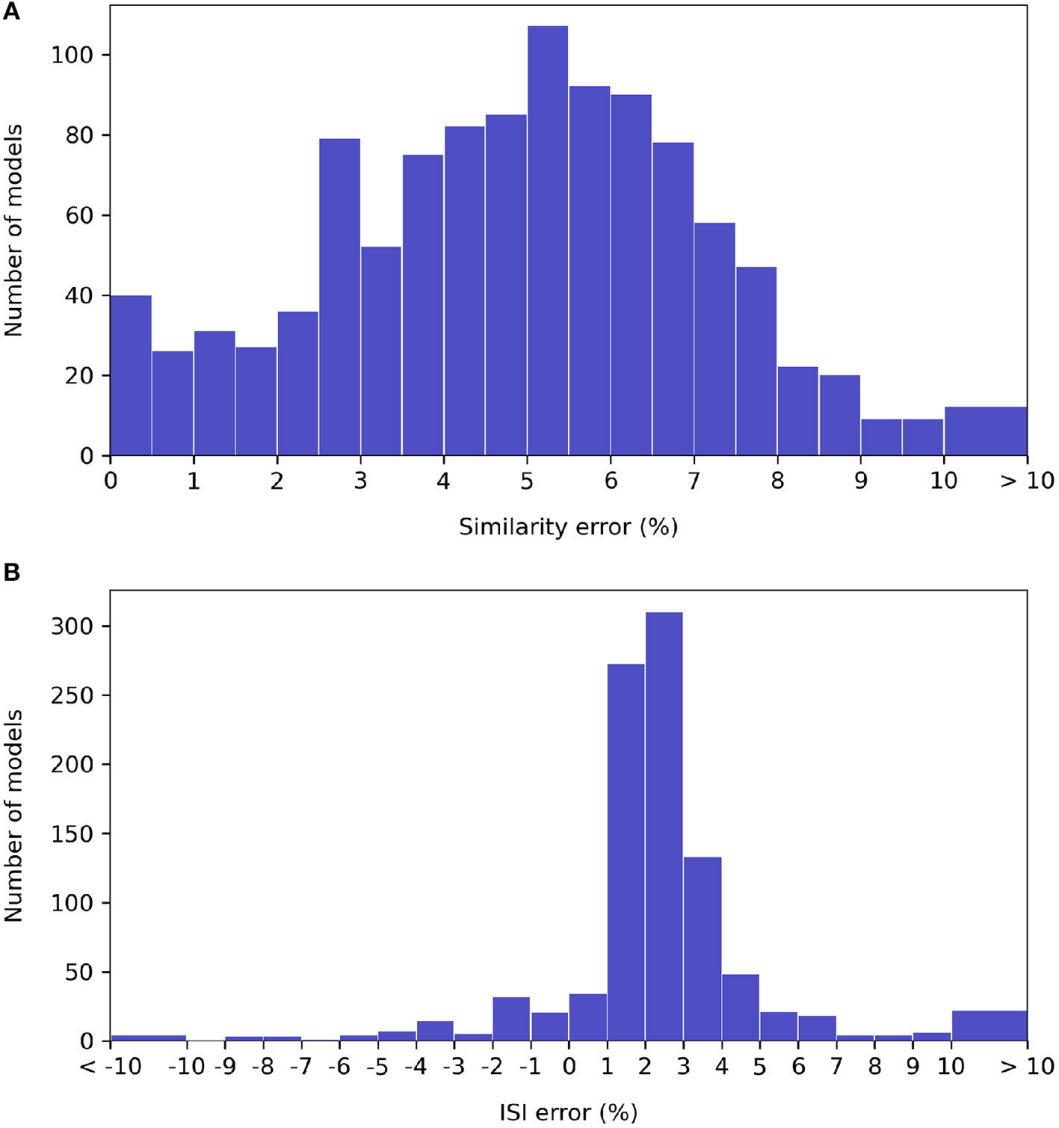
Histograms of relative error under the NeuroML-DB similarity **(A)** and inter-spike interval **(B)** accuracy metrics for each NeuroML-DB neuron model. The bins around the “ < -10” and “>10” limits include all models with more than 10% of discrepancy.

A histogram of waveform accuracy under each metric for the specified timestep, over all neuron types, is shown on Figure 7. (The models that either EDEN or jNeuroML could not simulate are excluded).

We observe that the EDEN's discrepancy against NEURON under the NeuroML-DB similarity metric is centered around 5%, and 99% of the models run under EDEN at <10% of waveform error. Under the inter-spike interval metric, EDEN's difference with NEURON is centered around +2.5%, with 90% of models having < ±5% difference and with 98% of models having < ±10% difference in mean inter-spike interval compared to NEURON.

Regarding error in the NML-DB metric, this is typically high for certain models with a high firing rate; as the waveforms get out of phase this metric drops sharply, even though the waveform of a single firing period has the same overall shape^5^. Regarding the discrepancies in firing period: We compared the mechanisms present on each neuron model with a high ISI difference between EDEN and NEURON. These neuron models do not share a distinct mechanism type, or other distinguishing commonality that could explain this; nonetheless, we note all these models originate from the Blue Brain Project collection and they showed a low firing rate under the protocol's clamp current. Since these neuron models emitted few spikes, the difference might be specific to the starting phase of regular firing, when induced by DC current.

The full set of results with accuracy metrics and waveform plots for each simulated model is available on Zenodo: https://zenodo.org/record/5526323.

#### 3.1.2. Evaluation on Neural Network Models

To assess EDEN's functionality when simulating networks, the result data from simulating the smaller versions of each network used in the performance benchmark on Section 3.2 were analyzed. Note that the enlarged versions of the GCL and cGOC models should not be used for functional analysis, because they have not been validated by the creators of the original models and they serve only as a computational benchmark.

**Figure 8:**
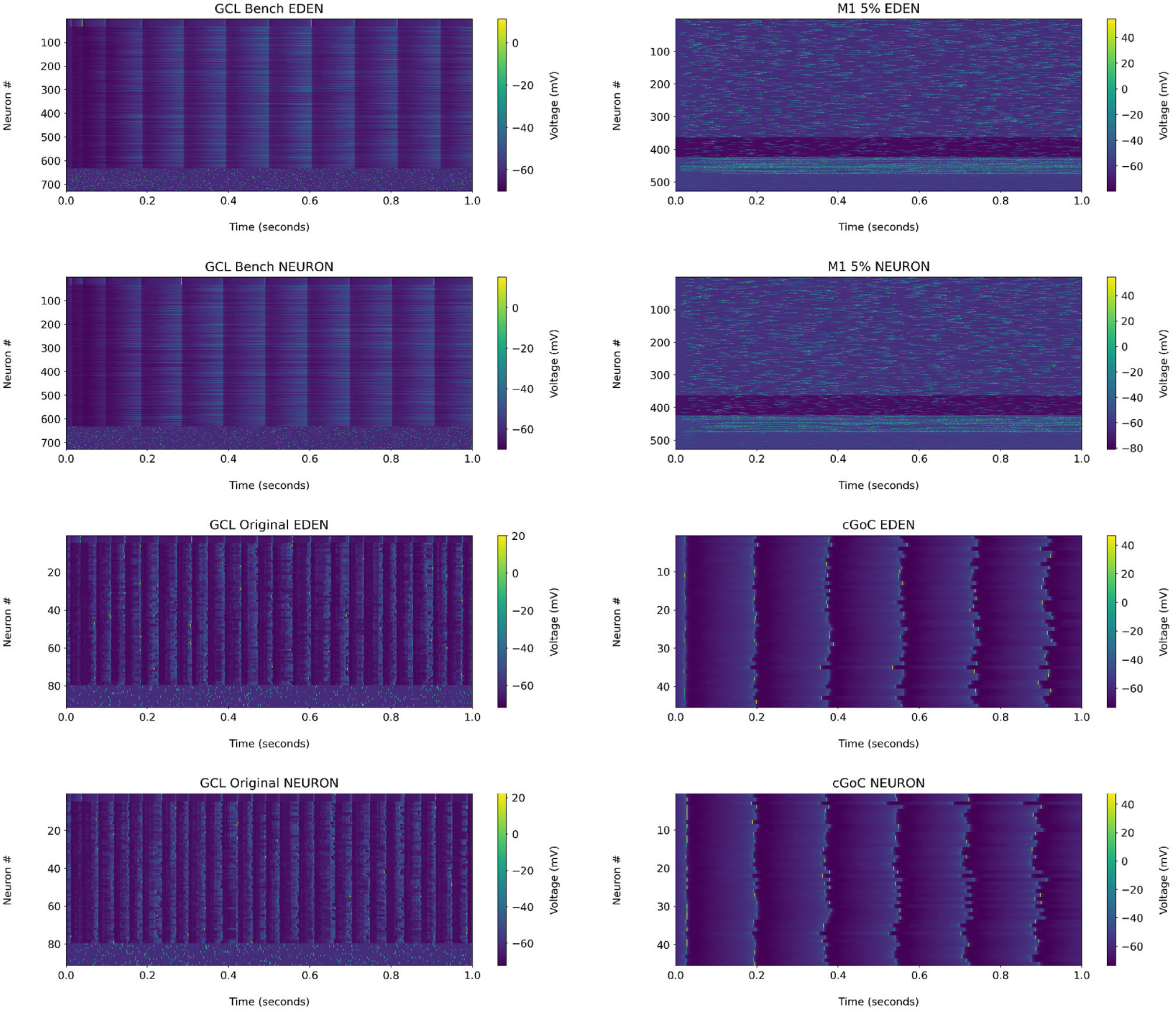
Raster plots for each network used in the performance benchmarks, when run on NEURON and EDEN. Note that the input stimuli are pseudo-randomly generated.

For reference, the raster plots for the networks analyzed in the following are shown in Figure 8. We observe that in the GCL model used for the benchmark, the granule cells did not generate action potentials under either NEURON or EDEN; a closer inspection of the voltage waveforms of these cells indicated that they are over-inhibited by the synapses. Therefore, we chose to apply the analysis on the original, smaller, single-compartment version of the network, which is also discussed in the Supplementary Material. The raster plot of this version of the network is also included there.

Since the networks are driven by random stimuli, the results cannot be compared directly as waveforms, but through network activity statistics. We employed the analysis methods proposed by Gutzen et al. (2018), who used them to compare the results generated by the SpiNNaker system and the original floating-point arithmetic based C code, for an Izhikevich cell network.

The measured metrics are: average firing rate (simply number of spikes divided by simulation time), local variation, mean inter-spike interval, correlation values of the binned spike trains with small and large temporal bins (metrics CC and RC respectively), and eigenvalues of the correlation matrix (computed by correlating the exact waveforms, in this work). For each of the metrics except for eigenvalues, the effect size between NEURON and EDEN's results is computed as Cohen's *d*, that is the difference of mean values of the distributions, divided by the pooled standard deviation of the two distributions. The 95% confidence interval for each effect size is calculated using the formula: $\pm 1.96\sqrt{\frac{N1+N2}{N1N2}+\frac{{\left(\text{effectSize}\right)}^{2}}{2\left(N1+N2-2\right)}}$

**Figure 9:**
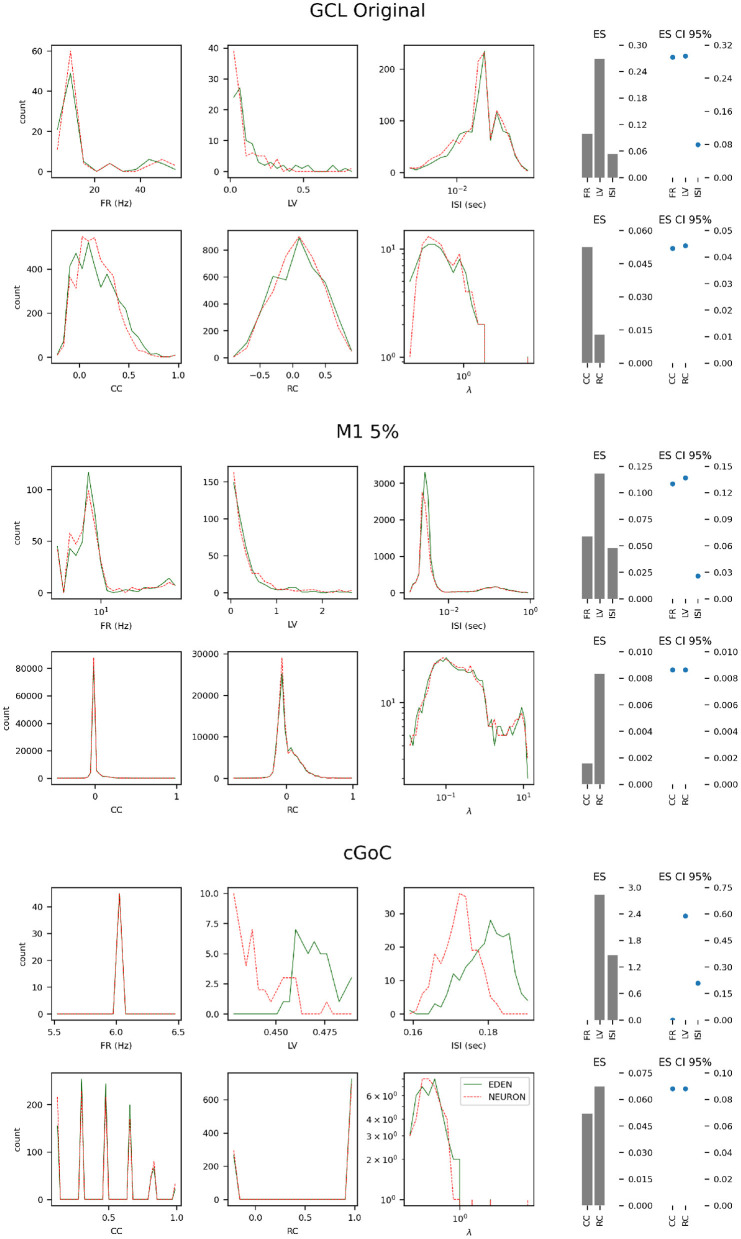
Histograms of neural activity metrics for each network used in the performance benchmarks. FR, firing rate; LV, local variation; ISI, inter-spike interval; CC, short-time firing correlation; RC, rate correlation; (**λ**), correlation eigenvalue. For each simulated network, the solid green line outlines the distribution of metric values when using EDEN, and the overlaid dashed red line outlines the distribution when using NEURON. On the right, the effect size (ES) and its confidence interval is shown for each applicable metric.

**Figure 10:**
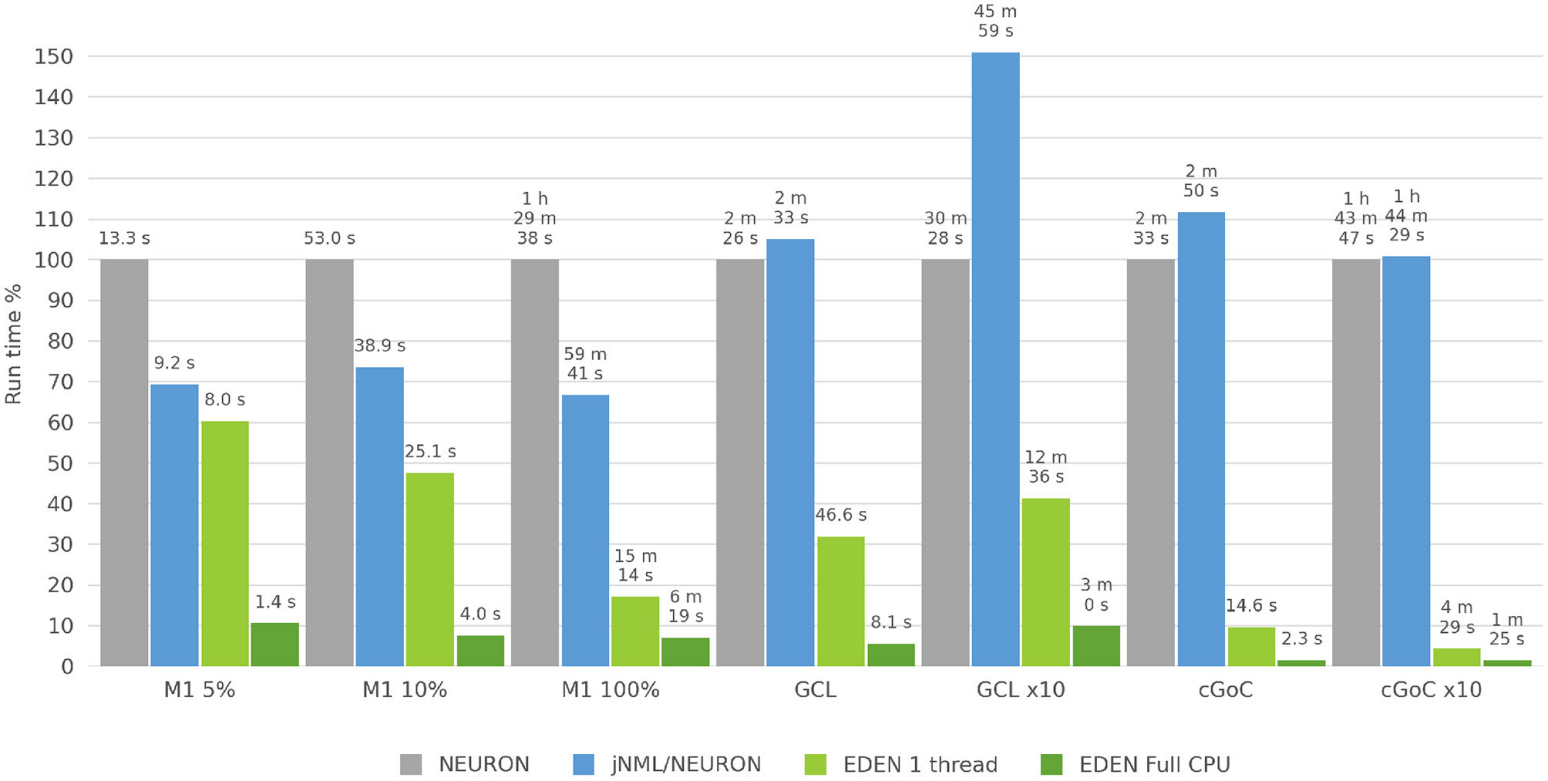
Run time for each neural network considered, for NEURON versus jNeuroML/NEURON and versus EDEN on one CPU thread and on all CPU threads. For each neural network, benchmark the bar height in the chart is normalized against NEURON's run time for that benchmark.

The resulting metrics for each network are shown on Figure 9. Presentation is similar to Figure 10 of the Gutzen et al. (2018) article.

For each of the networks, we observe the following:

- The results for the GCL network, we observe a slightly wider distribution of average firing rates in EDEN's results than in NEURON's, which is however not reflected in the inter-spike interval metric. In contrast with the other two networks, this one exhibits a wide range of neuron-pair correlation coefficients, both in fine time resolution and in rate correlation.
- The results for the M1 network are largely the same. This was expected, since NEURON and EDEN produce very similar results when simulating artificial cells (see also results from various OSB models in the Supplementary Material).
- The neurons of the cGOC network exhibit periodic, synchronized spiking. Thus all neurons exhibit the same estimated firing rate and are concentrated around specific values in fine temporal and rate-based correlation. Furthermore, the short-term and rate-based correlation indices are tightly clustered around specific values. There is a slight but clear difference between the simulators on the means of local variation and inter-spike interval; the effect size is very high because the variation in these metrics is very small across the neuron population (see the range of the in the LV and ISI plots).

In many cases, as the effect size is estimated to be low, the confidence interval for the monovariate metric s(firing rate, local variation, inter-spike interval) is determined by the small number of data points in the sample (i.e., neurons). Overall, the quantitative analysis indicates that our simulator succeeds in capturing the characteristics of simulated networks, much like NEURON does.

### 3.2. Computational Performance Analysis

#### 3.2.1. Overview

Beside flexibility in supported models, another distinguishing characteristic of neural simulators is speed. To evaluate the simulation speed EDEN offers we ran simulations of neural networks available in NeuroML literature, on a recent cost-effective desktop computer. We chose to run published neural networks over synthetic benchmarks, because:

- they have been used in practice, so they are concrete examples of what end users need; and
- existing models are usually the base for newer models, so the insights about the former do remain relevant.

Since the original neural networks were developed with the computing limitations of earlier years, these days they run comfortably in a desktop computer, using a minor fraction of system memory and within just a few minutes per run. (Unfortunately, new network models that do push the limits of present hardware, are still only available in heavily custom setups, that cannot be easily ported to another data format, simulator, or HPC cluster). To evaluate simulation performance for longer simulation run times, and more challenging neural network sizes, we also used enlarged versions of the original neural networks. This was possible, because the original networks were themselves procedurally generated, with parametric distributions of networks and synapses.

The neural networks that were run for performance evaluation are listed in Table 2, along with quantitative metrics for each case. Beside these quantitative metrics, there also are substantial qualitative differences between the models. These differences determine both the neural functions of each network, as well as the required computational effort to simulate each one.

**Table 2:** The simulated networks used for performance benchmarking.

**Simulation**	**Simulated** **time (s)**	**Steps**	**Compartments** **per neuron**	**Neurons**	**Total** **compartments**	**Total** **synapses**
GCL	1	100,000	1 ~ 4	728	2,624	7,958
GCL x10				7,280	26,240	79,825
M1 5%	1	20,000	1	527	527	15,469
M1 10%				1,065	1,065	61,538
M1 100%				10,734	10,734	5,032,223
CGoC	0.1	40,000	319	45	14,355	472
CGoC x10				450	143,550	5,410

#### 3.2.2. Simulated Networks

The neural network models used were the following, sourced from NeuroML-DB:

1. A multi-compartmental extension of the (Maex and Schutter, 1998) Cerebellar Granule Cell Layer (**GCL**) model (NeuroML-DB ID: NMLNT000001).
2. An Izhikevich cell-based, multiscale model of the mouse primary motor cortex (**M1**) (Dura-Bernal et al., 2017) (NeuroML-DB ID: NMLNT001656).
3. A model of the Golgi cell network in the input layer of the cerebellar cortex(**CGoC**), electrically coupled with gap junction (Vervaeke et al., 2010) (NeuroML-DB ID: NMLNT000070).

##### 3.2.2.1. The GCL Network

The GCL network is based on the Maex and Schutter (1998) model for the cerebellar granule cell layer, which includes granule cells, Golgi cells, and mossy-fiber cells. The designers of the GCL network benchmark extended the original GCL model to have multi-compartmental cells; in particular, the axons and parallel fibers of the granule cells are spatially detailed with four compartments per cell, and Golgi cells follow the ball-and-stick model, with 4 compartments per cell. The mossy-fiber cells are stimulated by Poisson randomly firing synapses and stimulate the granule cell population through AMPA and blocking NMDA synapses. The granule cells excite the Golgi cells through AMPA synapses, and the Golgi cells inhibit the granule-cell population through GABA_*A*_ synapses. We enlarged the original GCL network, by multiplying the population size by a factor of 10, and keeping the same per-neuron synapse density for the various projections. Thus, the total number of synapses was also 10 times the original.

##### 3.2.2.2. The M1 Network

The M1 network is an Izhikevich cell-based model of the mouse primary motor cortex, with various groups of cells intertwined across cortical depth. There are 13 groups of cells and four different sets of dynamics parameters among the cells. Each cell is stimulated by an external randomly firing synapse stimulus, and cells interact with each other through excitatory AMPA and NMDA synapses, and inhibitory GABA synapses. All synapses follow the stateless, double-exponential conductance model. This model is rather recent, so in its full size, it is computationally challenging enough to simulate, without enlarging it.

To better investigate performance characteristics, and evaluate performance at a model scale similar to the original GCL and CGoC networks, we generated two smaller versions of the M1 network, at “scale” values of 10 and 5%. Note that the model uses fixed probability connectivity for the various projections between populations, thus the number of synapses grows quadratically with the population size.

##### 3.2.2.3. The CGoC Network

The CGoC network models a small part (0.1 mm^3^) of the Golgi cell network, in the input layer of the mouse's cerebellar cortex. It was used in Vervaeke et al. (2010) to investigate the network behavior of Golgi cells, using experimental data. In this network, the neurons communicate with each other solely through gap junctions. Each cell also has 100 excitatory inputs in the form of randomly firing synapses, randomly distributed among apical dendrites.

Gap junctions have rarely been introduced in large network models in the past. This is not because they are absent from tissue, nor because their effects are negligible, but primarily because of their intense computational requirements. The continuous-time interaction between neurons that gap junctions effect requires a large amount of state data to be transferred to simulate each neuron in every step, while spike-based synapses need to only transfer the firing events between neurons, whenever they occur (rarely, compared to the number of simulation steps). As with the GCL network, we also enlarged this network, by making neuron count, synapse count and network volume 10 times the original.

#### 3.2.3. A Note on Numerical Methods & Performance

In the following, computational efficiency is compared between EDEN and NEURON (run under jNeuroML), for the same models. We notice a disparity in per-thread efficiency between EDEN and NEURON, and an immediate question is whether the difference is caused by differences in the simulators' numerical methods.

We noticed that, because of the MOD files, jNeuroML generates in the present version, most membrane mechanisms are simulated with the Forward-Euler integrator, as they are under EDEN as well^6^. The methods EDEN uses in these benchmark are explained in Section 2.5.5. The only clear difference in numerical methods between NEURON and EDEN is that EDEN uses single-precision arithmetic whereas NEURON uses double-precision arithmetic; but this is not enough to explain the observed disparity in simulation speed. Thus, we expect that our simulator's improved performance comes mostly from a more compact control flow, improved data locality, and reduced memory usage (since memory transfers also are a factor) than from pure numerical efficiency.

#### 3.2.4. Benchmark Results

The three neural networks described above—with network sizes, simulation time and time-steps as shown on Table 2—were run on a recent desktop PC, and simulation run time was measured in each case. The NEURON simulator was chosen as a baseline to compare simulation speed, because it is the predominant simulator for biophysically detailed, multi-compartmental neuron models. The models were run on NEURON, using both a direct-to-NEURON export of the networks as well as the HOC/MOD code that jNeuroML generates automatically. Although NeuroML2 models can be run on NEURON directly through the jNeuroML tool, there is no published information on the computational efficiency of simulations run through jNeuroML, compared to running hand-written NEURON code for the same model. By running both versions of the model, we compare EDEN's computational efficiency directly against NEURON, and also evaluate experimentally and publish the first data points on the computational efficiency of running neural networks on NEURON through jNeuroML.

All three networks used in the benchmarks were originally generated with a high-level model generation tool; this was neuroConstruct for the GCL and cGoC models, and NetPyNE for the M1 model. This fact also serves as an indication that model creators prefer to focus on the pure aspects of their models, than spend effort on the simulator-specific programming. We checked the implementations of the networks that these tools export for NEURON, and found that the implementations are as efficient as a modeler would reasonably write them to be.

- For the GCL and cGoC models, the HOC and MOD code was generated by neuroConstruct; however, we inspected the generated code and found that it is similar to how the HOC and MOD files are typically written in practice. The only difference is that the HOC statements to create the network are laid out as explicit lines, whereas on manual code loops and procedural (or file-based) generation would have been used to populate the network. However, once NEURON's run() command is run, the simulation is controlled by the hard-coded NEURON engine, save for the NMODL mechanisms; whose code, although machine generated, is straightforward and efficient. As explained below, the time to initialize each model is excluded from the measurements.
- The original M1 network was generated at runtime and loaded into NEURON through NetPyNE, which uses the simulator's Python API. The MOD file for the Izhikevich cell model was hand-written and supplied by the model creators, and the Exp2Syn mechanism for synapses is one of NEURON's built-in mechanisms. This use case is thus considered to be how the model is run directly on NEURON.

Although NEURON can employ multi-process parallelism on a network simulation, setting up a simulation for this requires non-trivial, simulator-specific programming code that is difficult to keep free of errors, and possibly changes in the model's MOD files to allow parallel processing. NetPyNE aims to remedy this but parallelizing a NEURON simulation still relies on non-trivial custom programming and care by the model creator. We explored ways to run NEURON in parallel using the existing NeuroML tooling, but none worked correctly for our models. Thus, NEURON was run on a single processing thread for all simulations; this represents the use case of running a NeuroML model on NEURON directly, without extensive NEURON-specific modifications. EDEN was also run on a single thread, allowing a direct comparison of intrinsic computational efficiency of the two simulators (that is, excluding EDEN's performance boost from automatic parallelization).

In this work, in order to focus on the pure computational efficiency of simulating the networks, we excluded the time needed to set up the model and to write result data from our measurements; we only measured the wall-clock time to run the model over the simulated time. EDEN's run time was measured both when using all CPU cores of the PC and when running on a single CPU thread. These two cases reflect different usage scenarios of the simulation workload: the first one occurs when an individual simulation has to be run and the second one when a large batch of simulations has to be run, as a group. The uses and the technical considerations for each case are explained below.

The first case is relevant when a neural network is simulated once, and its behavior is empirically assessed by the experimenter, who adjusts parameters interactively. This takes place in the first exploratory steps of development, when the experimenter is still deciding on the form and type of dynamics of the network. In this case, a single simulation has to be run as quickly as possible, using all available computational resources. Thus, EDEN uses all CPU cores simultaneously to run this simulation.

The second case is relevant after the network's form and model are determined (or candidates for a more extensive evaluation). In this case, the model's properties are explored, by varying its structural and physiological parameters across simulations, and measuring high-level metrics for the behavior of the network (such as type of firing activity and emergent correlations). To that end, a batch of up to thousands of simulations has to be run, in order to explore each individual point of the parameter space. Simulations can then run on a separate CPU thread each, in parallel. Some technical effort is required to distribute the runs of the batch among CPU cores but this technique also allows non-parallel simulators to use multi-core computers effectively. Even so, this kind of parallelism has its limitations. If parameter exploration is performed on a relatively large network, the high memory requirements may prevent launching as many model instances as CPU cores. In that case, it would be better to assign multiple CPU cores per simulation. Likewise, a non-uniform memory hierarchy (common in high-end compute nodes) could even make it more efficient to run fewer models simultaneously, with more cores assigned to each.

For all performance benchmarks, the machine used was a desktop PC, with an Intel i7-8700 3.2 Ghz CPU and 32 GB of 2133 MT/S DDR4 RAM. The CPU has six physical cores and can run up to 12 (hyper)threads simultaneously. The particular CPU was selected to reflect the typical, current-day system available on a researcher's desk–rather than what is available on a supercomputer setting, which requires substantial technical effort to use and is often not available for day-to-day experimentation. The OS used was Ubuntu Linux 18.04. NEURON, EDEN and the code generated by both at runtime were all compiled using the GNU C compiler, version 7.4.

The results for the performance benchmarks are shown in Table 3. For each simulation in Table 2, the time to run it is shown when running NEURON directly, NEURON through jNeuroML, EDEN on one CPU thread, and EDEN on the whole CPU. The corresponding speedup ratios for EDEN on a single thread and on all threads over NEURON are also shown on the table. Figure 10 visualizes the relative time to run each simulation with EDEN, using one CPU thread or the whole CPU, against the time to run the same simulation with NEURON.

**Table 3:** Measured run time for benchmarks for NEURON on 1 thread, jNeuroML/NEURON, EDEN on 1 thread, and EDEN using all CPU threads, and respective speedup ratios.

**Benchmark**	**NEURON** **run time (s)**	**jNML** **run time (s)**	**jNML** **speed ratio**	**EDEN run time (s)**		**EDEN speed ratio**	
				**1 thread**	**Full node**	**1 thread**	**Full node**
GCL	145.71	153.03	×0.95	46.55	8.07	×3.13	×18.05
GCL x10	1,828.18	2,758.91	×0.66	756.20	179.54	×2.42	×10.18
M1 5%	13.28	9.20	×1.44	8.00	1.41	×1.66	×9.46
M1 10%	52.99	38.93	×1.36	25.12	3.98	×2.11	×13.32
M1 100%	5,378.23	3,581.39	×1.50	914.17	378.74	×5.88	×14.20
CGoC	152.69	170.36	×0.90	14.64	2.33	×10.43	×65.45
CGoC x10	6,227.36	6,269.13	×0.99	268.75	85.22	×23.17	×73.07

We observe that EDEN largely outperforms NEURON while running on a single CPU thread, and even more so when the network is simulated across all threads of the CPU. This is because EDEN was designed from the start to achieve high computational performance, especially when running complex, biophysically detailed neurons. In the following, we will comment on the performance characteristics demonstrated when running each specific neural network, and reiterate the network's properties that affect computational performance.

The GCL network comprises biophysically detailed cells, with a very small number of compartments per cell. In this case, EDEN generates fully simplified code kernels for each neuron type; the code to simulate each individual compartment is laid out as an explicit, flat sequence of arithmetic operations.

When running the original GCL network, EDEN runs at 3.1 × the speed of NEURON, using one CPU thread. This level of speedup over NEURON applies when running a batch of many small simulations; in which case, each simulation is run on a single CPU thread for best results. By utilizing all six cores of the CPU, simulation speed further improves around six times, for a total of 18 × the speed of NEURON. This shows that when a single simulation has to be run at maximum speed, EDEN can automatically, efficiently parallelize the computational work across multiple processor cores to run faster. For the enlarged version of the network, single-thread speedup using EDEN is less, to 2.4 × the simulation speed of NEURON. Speed improves by using all threads, but the total improvement in speed vs. running NEURON is not as great as when running the smaller, original-size model (×10.18 total, compared to ×27.1 previously). It could be that the processor's data transfer speed decreases with model size, and limits computational throughput; however, the fact that jNeuroML runs significantly slower than NEURON for this network could indicate that there is an inefficiency involved in interpreting the NeuroML2 version of the model. At any rate, this relative slowdown of the NeuroML-based simulators warrants further investigation.

The M1 network comprises Izhikevich-type artificial cells, with dense synaptic connectivity between the neurons. In this case, each neuron's internal model is one Izhikevich-cell mechanism; EDEN generates a simplified code kernel, that simulates the neuron's internal dynamics and synaptic interaction. When running the 5% version of the network, EDEN on a single CPU thread runs at 1.7 times the speed of NEURON, and using all cores it runs at 9.5 times the speed of NEURON. Running the larger 10% network, these performance ratios increase to 2.1 × and 13.3 ×, respectively. Finally, when running the full-sized version of the network, EDEN on one CPU thread runs at 5.9x the speed of NEURON, and using all cores it runs at 14.2 × the speed of NEURON. For the reduced-size versions of the network, EDEN still runs faster than NEURON, but not by as much as when running the full-sized version. This might be because the amount of computations and data involving these simplified neurons is smaller (also due to the smaller number of synapses per cell, in the M1 network), which increases the effect of parallelization overhead for EDEN. For the full-sized network, EDEN's relative performance improves steadily. Another interesting observation is that all sizes of the M1 network run significantly faster as NeuroML models under jNeuroML/NEURON than as the original NetPyNE/NEURON model. We speculate that this is because of the MOD file describing the neurons; the original hand-written one contains many additional features, calculations and WATCH statements which are not used in this model. Compared to the original MOD file, the one that jNeuroML generates automatically is quite simpler.

Networks solely consisting of point neurons can already be run with high computational performance, on specialized simulators like NEST. However, there is the important class of hybrid SNNs (Lytton and Hines, 2004) that mixes physiologically-detailed and artificial cells according to the focus of each model. Such networks have to be run with general-purpose neural simulators, that support both types of neuron, which then need to run in tandem. By demonstrating a consistent high speedup factor even for artificial-cell networks that are not its main target, EDEN shows that it can run hybrid neural networks at a greatly increased speed, without running into performance problems. For pure artificial-cell networks, EDEN is still relevant for modifications that depart from the commonly supported models, or take a lot of effort to set up on high-performance artificial-cell simulators (e.g., require modifying the simulator's source code to extend model support).

The CGoC network is made up of Golgi cells, which are modeled with hundreds of physiological compartments. Since these cells have too many compartments to apply a flat-code representation per cell type, as was done for the GCL network, EDEN works differently in this case. For each type of cell, the compartments comprising it are grouped according to the set of physiological mechanisms that they contain. This way, one code kernel is generated for simulating each different type of compartment. Then, all compartments of the same type are simulated as a group using a loop over the same code. After computing the internal dynamics for each compartment, the interactions between the compartments, such as the cable equations, are also computed to complete the time-step. We notice that, when running either the original or the 10x-enlarged version of the CGoC network, EDEN exhibits a spectacular increase in simulation speed compared to NEURON. When running the original-sized network, the relative simulation speed over NEURON is 10.4 × using one thread, and 65.5 × using all threads. In wall-clock terms, this means that a simulation that used to take two and a half minutes to run with NEURON, takes 14.6 s with EDEN in batch mode, and 2.3 s with EDEN in single-simulation mode. When running the 10x-enlarged version of the network, the relative simulation speed using NEURON is 23.2 × when using one thread, and 73.1 × when using all threads. In this case, wall-clock run times are 1 h 44 min to run with NEURON, vs. 4 min 29 s with EDEN in batch mode and 1 min 25 s with EDEN in single-simulation mode. The significant improvement in speedup that EDEN exhibits when running the cGoC network vs. the other two networks indicates that the current implementation is best suited for large populations of highly detailed neurons.

## 4. Discussion

### 4.1. Current Neural-Simulator Challenges

Through the process of developing EDEN and our involvement with the existing neural-modeling literature, tools and practices, we realized the urgent need for *standards* in brain modeling and *reproducibility* between simulators.

From the perspective of a computer engineer, there is an enormous learning curve in designing simulators for biophysically-detailed neural networks. The technical know-how on handling the differential equations of neural physiology is scattered across past publications and program source code and, even then, is rarely mentioned by name. A modeling standard could help form a compendium of all the mathematical concerns that affect simulator design, and would allow neuroscientists and engineers to co-operate efficiently.

As mentioned in Section 1, when working with highly detailed neural networks, swapping among different simulators during experimentation would take an impractical amount of effort. This is one of the reasons why there are so few inter-simulator comparisons of the same model in *in silico* neuroscience literature and why they usually only concern porting a custom simulation code to, or from, a general-purpose simulator. A standardized, interoperable description for models would remove this major obstacle and enable cross-simulator evaluation. There do exist software that can algorithmically generate neural networks and run them on different simulators [examples are PyNN (Davison et al., 2009), and also neuroConstruct *via* NeuroML export and jNeuroML]. The problem with them is that the model-building “recipes” they support are few and basic. However, model creators often use highly custom methods to construct their networks, which prevents them from using the multi-simulator capability of tools to save programming effort. A solution to this conundrum may be to use an unambiguous description for generated neural networks, such as NeuroMLv2; then, model creators still have to convert the networks that their custom methods generate to the common description, but multi-simulator capability is much easier to implement since the network to simulate is described explicitly. Still, this approach allows combining all types of network-building recipes with all simulation platforms, without extra programming effort for each combination. The related field of systems biology reveals a success story in the CellML (Cuellar et al., 2003) and SBML (Hucka et al., 2003) standards; however, those standards are still not sufficient for capturing modern networks of multi-compartment neurons.

Another important aspect of upcoming neuroscience projects is *multiscale modeling*; that is, studying a neural structure across multiple levels of modeling detail. Since this often involves many different simulators of different model types, it only becomes practical by adopting extensive standards that capture not only the different models but also the results of the simulation at each level. This is necessary in order to reconcile and investigate the different scales of modeling without writing fully custom, one-off code for each case.

The integrated TVB modeling platform (Sanz Leon et al., 2013) is currently the leading tool for multiscale brain modeling and features a complete methodology for integration of macroscopic neuroimaging data into models. However, this methodology is mostly designed around the specific TVB platform; there is effort to co-simulate with the NEST simulator specifically, but it is still at an ad-hoc, proof-of-concept stage.

Besides standards, we also advocate for a more rigorous *integration* of the various simulators with neuroscientific as well as general (e.g., Python/Jupyter) workflows, which will speed up experimental setup and enable seamless transfer of simulation results across different platforms. This may sound obvious but it is in fact a crucial element for real-world quick adoption and utilization of this ensemble of platforms. NEURON, BRIAN2, GeNN, and Arbor have already caught on to this need; that is why they all natively support a Python interface, alternative to their own custom languages (BRIAN2 itself is Python-native). EDEN already offers such integration through its pyNeuroML-compatible Python bindings^7^ and interoperability with the existing NeuroML tooling infrastructure before and after the simulation stage.

Regarding the NeuroML community, it is important to stress the usefulness of providing simulation files along with the published model descriptions. This is important not only to fully record the published experiments but also to be able to reproduce the experiments and cross-validate the results on multiple simulators. To illustrate, we tried to evaluate EDEN on as many NeuroML networks as possible but were only able to find five individual, non-trivial network models in the entire NeuroML-DB—and important simulation parameters such as duration, time-step size, and recording probes were only available in the original code repositories outside NeuroML-DB.

Finally, from an HPC perspective, the large-network simulation needs of modern researchers call for the use of computer clusters. However existing simulators either offer partial support for clusters or require advanced programming from the end user to work. Automatic, complete support for clusters must therefore be a development priority, which the simulator designers are best suited to address. EDEN offers such built-in automation and will continue improving on its performance.

### 4.2. The EDEN Potential and Next Steps

The evaluation presented makes it abundantly clear that EDEN delivers on its triple mission toward high performance, high model generality and high usability. This first version of EDEN was focused on ensuring that all kinds of NeuroML models are supported, rather than optimizing the performance of a limited subset thereof. Thus, the performance results seen in this work form a minimum guaranteed baseline of performance, on top of which future improvements can boost performance even further.

Even so, we showed that this performance baseline provides, for real-world neural networks drawn from NeuroML-DB, a speedup ratio over NEURON of 2~23 × per CPU thread and 9~73 × in total, on an ordinary desktop PC. We also demonstrated that no technical expertise is required for deploying and parallelizing the simulations of small and large networks alike, which presents a great incentive for the quick adoption of EDEN by the neuroscientific community.

All its achievements notwithstanding, EDEN is far from a concluded simulator. Our future plans involve work in various directions. Below, we enumerate a few crucial ones:

1. Validate further the EDEN architecture through integrating existing, best-in-class code kernels from the community for special cases (Kasap and van Opstal, 2018; Miedema et al., 2020). Characterize performance etc. on various types of neural networks so as to determine further performance margins.
2. Boost the EDEN general-purpose backend by porting it to accelerator hardware, e.g., on GPUs and graph processors. Employ graph-theory methods for problem mapping in order to deploy EDEN on heterogeneous (e.g., CPU-GPU) platforms and reduce communication overheads.
3. Study the structure and communication patterns of spiking neural network models used in practice, and develop sophisticated strategies to map large simulated networks to computer clusters most efficiently.
4. Add further extensions to EDEN for high-end HPC application, such as support for the SONATA data format and for simulation checkpointing.
5. Research and refine innovative numerical integrators, to improve computational parallelism and maintain numerical accuracy on challenges like cable equations and kinetic schemes.
6. Evaluate and propose extensions to EDEN and NeuroML that enable direct interfacing with arbitrary data sources such as video stimuli, simulated environments to allow training experiments, and dynamic clamps for hybrid experimentation.

## 5. Conclusion

The large scale, fast pace and ample diversity of *in silico* neuroscience necessitates simulation platforms that offer high computational performance alloyed with reproducibility, low complexity in model description and a wide range of supported mechanisms. To those ends, we have developed EDEN, a novel neural simulator that natively supports the entire NeuroML v2 standard, manages the simulation's technical details as well as multi-node and multi-core cluster resources automatically, and offers computational performance without precedent in the scope of general-purpose neural simulators.

## Data Availability Statement

The datasets presented in this study can be found in online repositories. The source code of the EDEN simulator is available on GitLab, through the URL: https://gitlab.com/neurocomputing-lab/Inferior_OliveEMC/eden. The benchmark files and scripts to reproduce the figures of the paper are available on Zenodo, accession number 5526323: https://zenodo.org/record/5526323.

## Author Contributions

SP designed and developed the EDEN simulator and conducted the experiments. HS was the technical manager of the simulator project and designed the experiments. MN provided guidelines for usability and for numerical issues of neural simulation. MN, CS, and DS conceived and supervised the simulator project. All authors contributed to the article, edited and wrote the manuscript, and approved the submitted version.

## Funding

This research was supported by the European Commission Horizon2020 Framework Programme Projects EXA2PRO (Grant Agreement No. 801015) and EuroEXA (Grant Agreement No. 754337).

## Conflict of Interest

The authors declare that the research was conducted in the absence of any commercial or financial relationships that could be construed as a potential conflict of interest.

## Publisher's Note

All claims expressed in this article are solely those of the authors and do not necessarily represent those of their affiliated organizations, or those of the publisher, the editors and the reviewers. Any product that may be evaluated in this article, or claim that may be made by its manufacturer, is not guaranteed or endorsed by the publisher.

## Footnotes

^1^Code generation as a general technique is prevalent in high-performance neural simulators—see Blundell et al. (2018)—but these simulators either analyse neuron models at a shallower level than our work does, or they support a narrow subset of neuron models, as we explain in the following.

^2^Neural networks with mixed populations of both artificial and biophysically modeled neurons.

^3^Common NeuroML-compatible model-generation tools: NetPyNE (Dura-Bernal et al., 2019), neuroConstruct (Gleeson et al., 2007), NeuroMLlite (https://github.com/NeuroML/NeuroMLlite).

^4^Polymorphic means that it adapts to the neuron's structure, instead of handling just one type.

^5^This effect had been discounted in the evaluation method used in the Rallpacks (Bhalla et al., 1992), by linearly distorting the waveforms of inter-spike intervals to have the same nominal duration for both simulators, before comparing.

^6^With the exception of Markov gates for ion channels. In that specific case, the “sparse” method is employed by jNeuroML, but the kinetic scheme that is present in cGoC cells has too few state variables to affect overall run time compared to using a simpler method.

^7^ https://pypi.org/project/eden-simulator/
