# Supplementary material for: EDEN: A High-Performance, General-Purpose, NeuroML-Based Neural Simulator
Source: Front Neuroinform. 2022 May 20;16:724336. doi: 10.3389/fninf.2022.724336 (PMC9167055; doi:10.3389/fninf.2022.724336)
Supplement: Supplementary file 1 [file Data_Sheet_1.pdf]

Supplementary Material

1 EVALUATION OF FUNCTIONAL CORRECTNESS THROUGH MISCELLANEOUS OPENSOURCEBRAIN MODELS

In addition to the regular evaluation methods used in the ”Evaluation of functional correctness” of the paper, in this section we illustrate various common features of neural activity, through NeuroML models available on the OpenSourceBrain code project repository.

A problem we encountered while reviewing this set of NeuroML models was to gather simulation configurations that fully represent the models of the original studies, and are hence useful to assess simulation fidelity. A model’s simulation configuration includes the network and stimulus setup, simulation duration and appropriate timestep, and the set of variables to be recorded. Even when a model is faithfully ported to NeuroML, the simulation configurations that come with it are often only simple tests of some components; a simulation configuration that captures the full model may be missing. Still, this is understandable when the original model needs custom simulator-specific code to set up, as it often happens. More than just having incomplete simulation configurations, some models are only partially implemented - a NeuroML model may, for example, originate from a neuron tracing project and thus include detailed morphology, yet lack active mechanisms which are necessary for a meaningful biophysical simulation. This is also evident in some of the following figures: while some of them show regularly spiking neurons, others show just a transient response to initialisation. Setting up a new simulation configuration for each of these models in a way that is appropriate for each is, unfortunately, beyond the scope of this research group.

With these precautions in mind, there still is value in examining the results of some provided simulation configurations. In the following, we selected a set of models with associated simulation configurations, to show a sample of what result data a user may get by the existing simulation configurations, and to illustrate finer neural features that are not considered in the more regular evaluation methods of the previous sections. Such features are the spatial distribution of membrane voltage over time, the gate variables of ion channels and the internal state variables of artificial cells; due to their functional and physical diversity, similarity between simulations of these variables cannot be assessed through a single protocol. For example, the propagation of an action potential throughout a neuron is difficult to assess among neuron models, since they typically have much different morphology and function; the state variables of active mechanisms are rarely comparable among different types.

For these reasons, we refrained from quantifying the similarity of EDEN’s and NEURON’s results in this section. This would only be appropriate if we had appropriate simulation configurations available; since we did have these for the models in the previous sections, we did apply rigorous mathematical analysis of the results then.

The specific models in use were gathered from the Open Source Brain code repository; the models’ summaries can be found in Table S1. The types of models are further described below.

In each case, EDEN’s simulation results were checked against the ones produced by NEURON for the same model. The line plots and analogue raster plots in the following figures show the results for each of the simulations run, for the two simulators. Line plots use specific colours for each state variable recorded;

Simulation	Type	Compartments	Network
Izhikevich 2007	Abstract	1	no
FitzHugh-Nagumo	Abstract	1	no
Moris-Lecar	Simplified biophysical	1	no
Hindmarsh-Rose	Simplified biophysical	1	no
MainenEtAl simplified	Extended HH	1	no
Amacrine	Passive	2105	no
Ferrante 2009	Extended HH	1091	no
MouseLight AA0173	Classic HH	850	no
OSB GPUshowcase	Linear I&F	1	10 cells
Maex et al. GCL	Extended HH	1	90 cells

Table S1. Features of the simulations used to test EDEN's functional correctness..

they use a darker dotted variant for the trajectories NEURON generates, and overlay a paler solid variant for the ones EDEN does. When the results match, the lines coincide.

In Figure S1, we show the simulated waveforms for a few common types of simplified cells, namely, the modified Izhikevich model (Izhikevich, 2007), Morris-Lecar (Morris and Lecar, 1981), Hindmarsh-Rose (Hindmarsh et al., 1984) and FitzHugh-Nagumo (FitzHugh, 1961) models. The first two are abstract cell models, in that their state variables (other than designated membrane voltage) have no physiological meaning, whereas the latter two are simplified biophysical models, where all state variables represent biophysical quantities, and they have dynamics that are less computationally challenging than those of the extended Hodgkin-Huxley class of biophysical models. In each model, the neuron receives a DC current input, which induces a periodic spiking pattern. In these plots, the trajectories produced by EDEN are virtually the same as the ones produced by NEURON. Usually artificial cells have slower, less stiff dynamics compared to physiological formulations; so, for that type of neurons, the simulations may not be so sensitive to the choice of numerical integrator.

Figure S2A shows simulation results for another kind of point neuron. This is a pyramidal cell model from (Mainen et al., 1995), but the spatial details of the neuron have been reduced to a single-compartment description. Stimulus sources were not given in the OSB-provided simulation configuration, so the neuron fires a transient spike and quiesces.

Figure S2B shows simulation results, for a single Dentate Gyrus Granule cell, as described in (Ferrante et al., 2009). This simulation tracks membrane potential for the soma of the neuron, although the neuron consists of 1091 anatomical compartments. We observe that a slight difference in timing occurs between the spike trains generated by NEURON and EDEN. However, this difference is much smaller than a spike period and evolves over a time course of 100 milliseconds. Due to the sensitivity inherent in spiking-neuron models, this small delay is enough to allow or inhibit the last spike, the moment when input stimulus stops.

Figure S2C shows simulation results, for a passive mouse-retina amacrine cell (Chen and Chiao, 2014). The output shows the effect of DC-current probes, placed in different places on the neuron. This model is based on the reconstructed morphology of the neuron, and physiological data are not available. Thus the NeuroML model includes only passive electrical leakage on the membrane, and transient DC clamps that

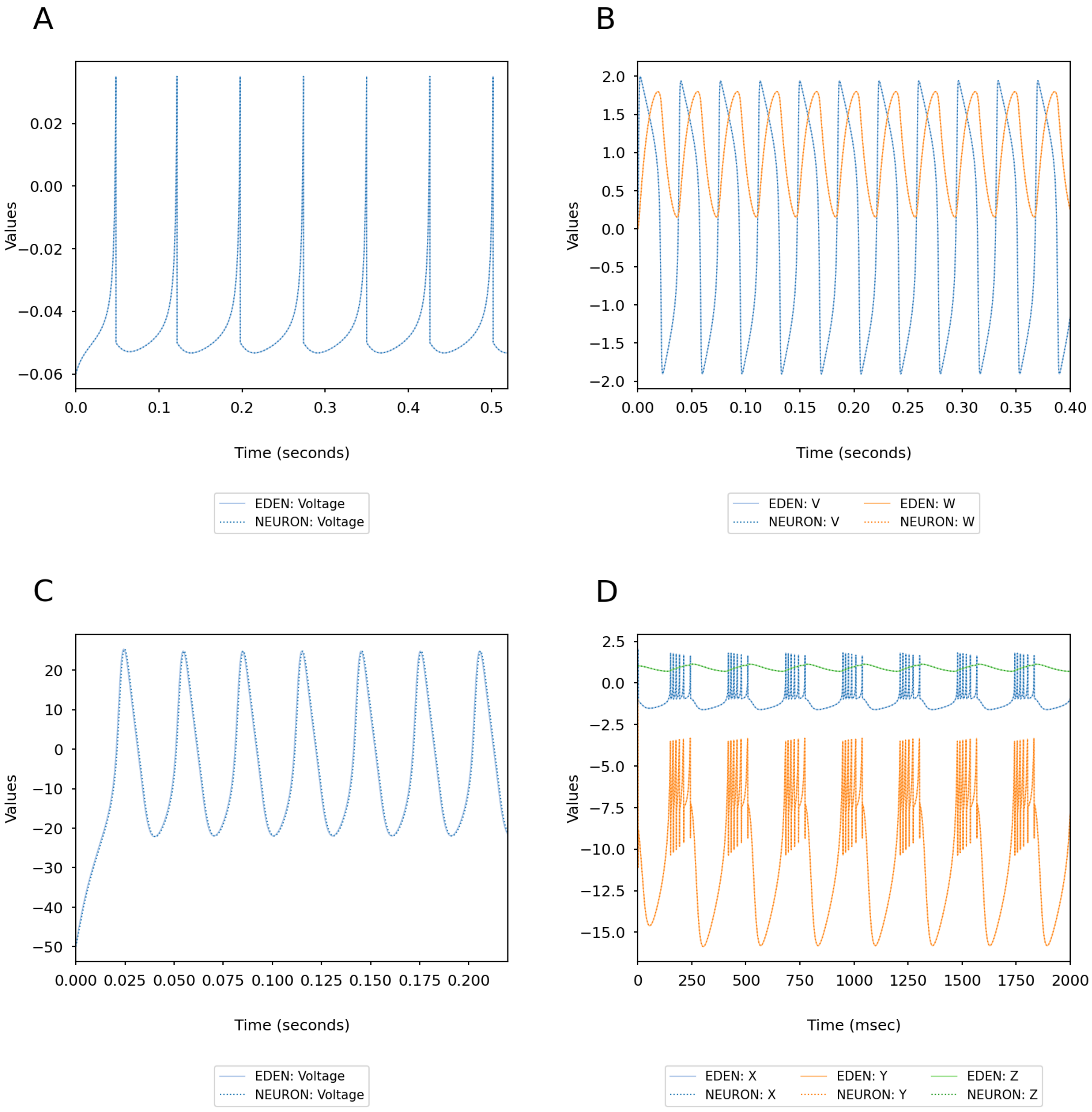

Figure S1. Validation results for various point-neuron types. The trajectories produced by NEURON and EDEN are plotted, with EDEN's trajectories overlaid on NEURON's for each model: Izhikevich 2007 model **(A)**; FitzHugh-Nagumo model **(B)**; Morris-Lecar model **(C)**; Hindmarsh-Rose model **(D)**. Voltage is shown for all models, and the internal state variables of the models are additionally shown in **(B)** and **(D)**.

operate at different points in time on different sites; the clamped sites are the soma and segments 500, 1000, 1500 and 2000 in sequence. Here, we can see that there are small steady-state variations in membrane potential between NEURON and EDEN, near the simulation site that has an active clamp each time. Since only membrane leak dynamics are present in the cell, and NEURON and EDEN use the same discretisation

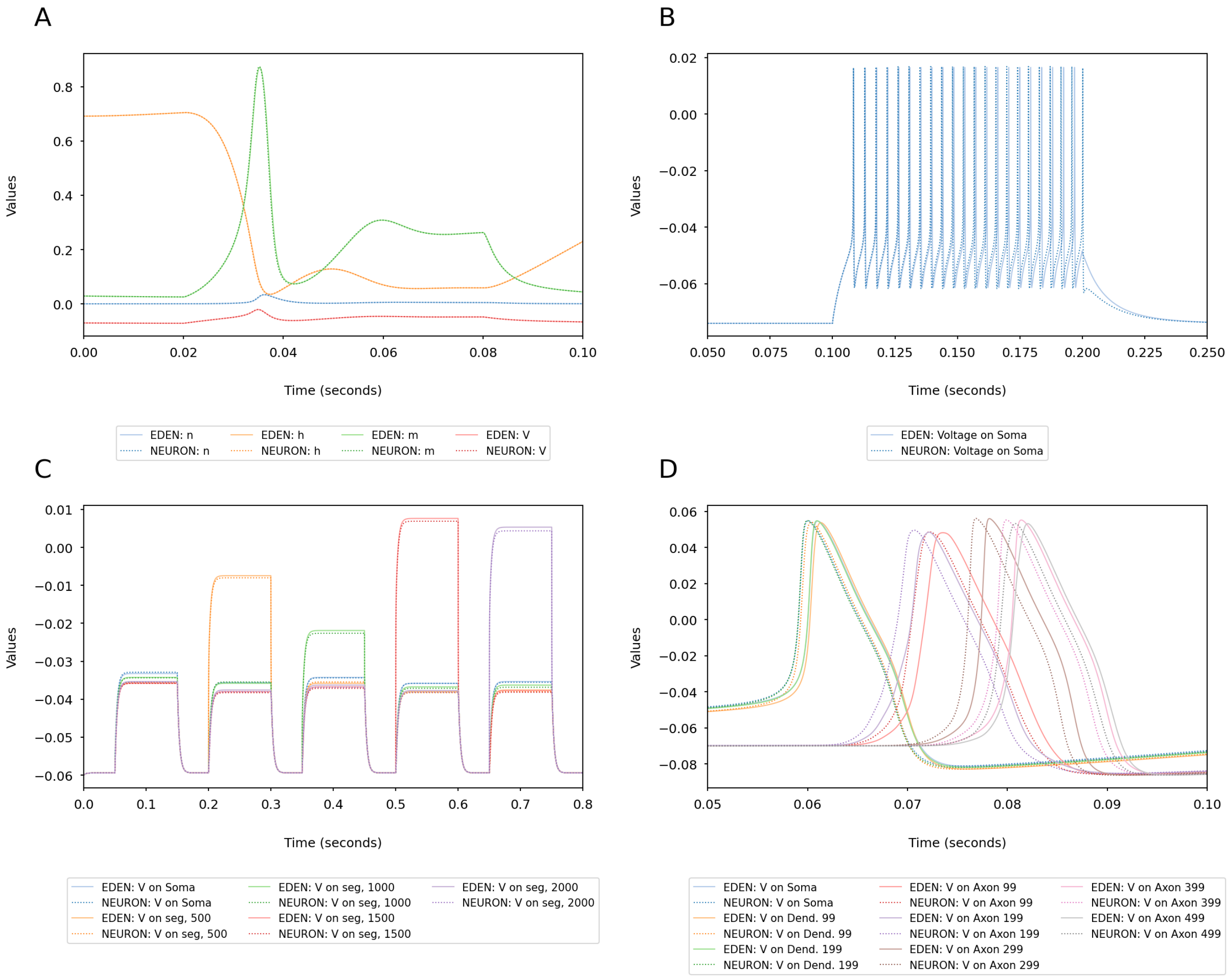

Figure S2. Validation results for various multi-compartmental neuron types. The trajectories produced by NEURON and EDEN are plotted, with EDEN's overlaid on NEURON's for each model: Mainen et al. 1995 single-compartment version model **(A)**; Ferrante 2009 Dentate Gyrus granule cell model **(B)**; Amacrine passive model **(C)**; MouseLight AA0289 model **(D)**. In **(A)**, the gate variables of the ion channels present on the soma are shown, along with membrane voltage on the soma; in the other subfigures, membrane voltage is shown for various sites on the neurons. In **(B)** and **(D)**, the initial and final time span where the neurons were resting has been trimmed out to enhance clarity in the active period.

method, we expect the discrepancies to be caused by the different numerical methods employed. Even though the model is not biophysically realistic, it has uncovered a minor numerical discrepancy between NEURON and EDEN while being very simple.

In Figure S2D, we see the simulation output for NEURON and EDEN on a reconstructed cell (number AA0173) from the Janelia MouseLight project (Economo et al., 2016). Since, again, only the passive morphology of the neuron was available, active cell models were produced by adding classical HH ion channels, uniformly distributed across the cell. In this simulation, we observe an action potential caused by a DC current probe on the soma, originating in it and travelling outward along the dendrites.

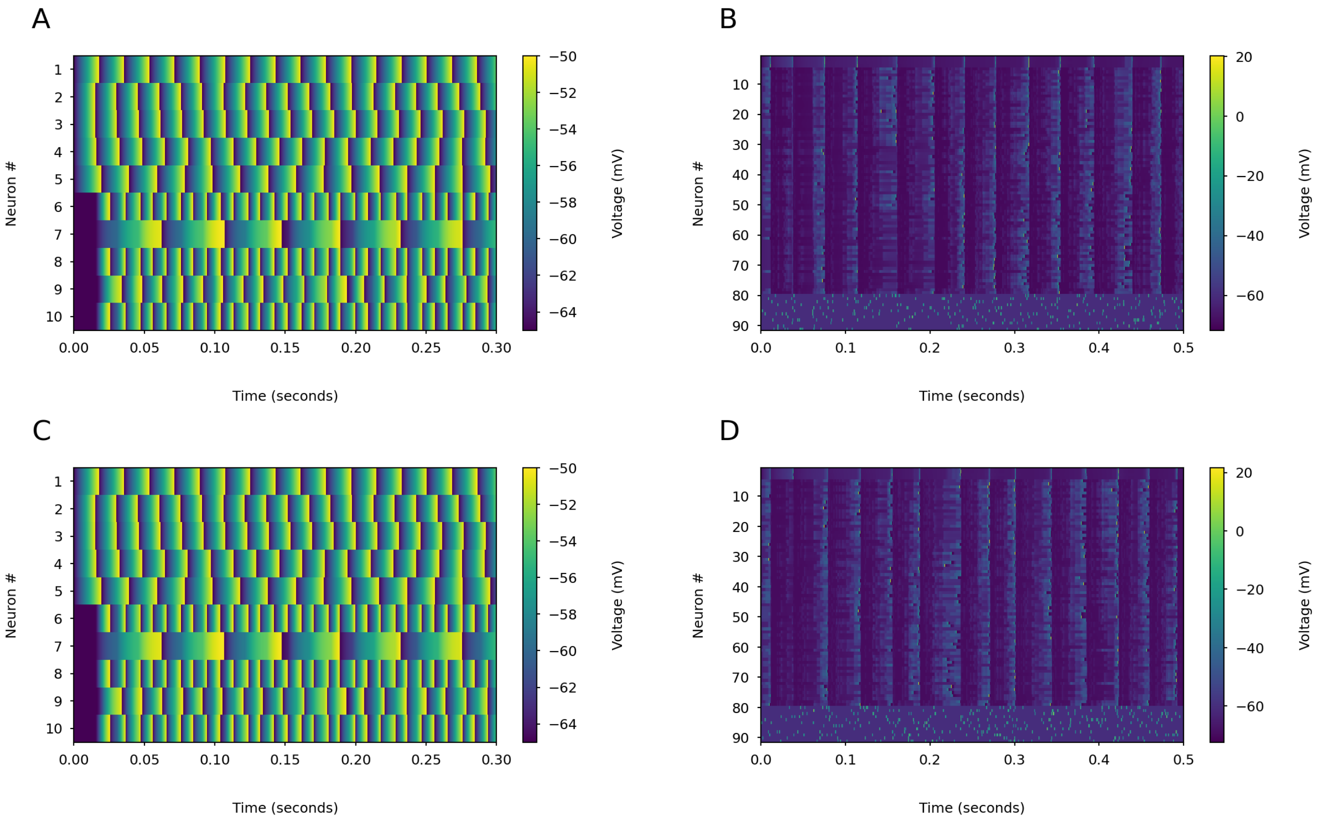

Figure S3. Validation results for the LIF and GCL networks. The membrane voltage trajectories produced by NEURON and EDEN are shown in analogue rasters, with NEURON's results on top for each model: LIF model **(A)** and GCL model **(B)**; and EDEN's results below, for the same networks **(C)**, **(D)**.

Figure S3 shows the analogue raster plots for two networks of neurons. The plots show the soma potential of the neurons in the network over time, with the horizontal axis representing time and the vertical axis representing neuron numbers. One network, shown on Figure S3 (A and C) consists of linear integrate-and-fire neurons, connected with double-exponential conductance synapses. It is a test network that is provided by OSB as a target for simulators on accelerator platforms. Since there is no random-variable component in this simulation, its results are deterministic. The results of the two simulators are virtually identical, just like in the previously shown simulations of isolated artificial cells.

The other network shown on Figures S3 (B and D) contains three interacting populations of physiologically-modelled point neurons, as per the cerebellar granule-cell layer model of Maex et al. (Maex and Schutter, 1998). The inputs provided to this cell are stochastic (double-exponential firing synapses, triggered by independent Poisson spike sources).

We observe synchronised firing in the group of Golgi cells (neurons 1 to 4), a more variable, roughly synchronous firing pattern in the group of granule cells (neurons 5 to 78) and random individual firing for the group of mossy fiber cells (neurons 79 to 90). The synchronised firing fronts for the entire network are not periodic. In fact, the synchronous and individual cell firing varies among runs with different randomisation seeds, on either NEURON or EDEN. Hence, results from any two runs of the model can only be compared through indirect metrics, even then the same simulator is used. However, we observe that the model exhibits similar activity patterns on both NEURON and EDEN. (Note that the shown raster

plots have a fine temporal resolution; the seemingly flat-coloured voltage 'boxes' that are visible are due to sub-threshold excitatory post-synaptic potentials, and their width in time represents the duration of the potentials.)

In conclusion, the above qualitative tests provide an insight on how close EDEN's simulation results are to the ones produced by NEURON, for various cases of neural models. In the case of point neurons, the results produced by both simulators are virtually the same. In the case of multi-compartment cells, the differences in simulation results are minor, and presumably caused by the different numerical integration techniques the simulators employ.

REFERENCES

Chen, Y.-P. and Chiao, C.-C. (2014). Spatial distribution of excitatory synapses on the dendrites of ganglion cells in the mouse retina. *PLoS ONE* 9, e86159. doi:10.1371/journal.pone.0086159

Economo, M. N., Clack, N. G., Lavis, L. D., Gerfen, C. R., Svoboda, K., Myers, E. W., et al. (2016). A platform for brain-wide imaging and reconstruction of individual neurons. *eLife* 5. doi:10.7554/elife.10566

Ferrante, M., Migliore, M., and Ascoli, G. A. (2009). Feed-forward inhibition as a buffer of the neuronal input-output relation. *Proceedings of the National Academy of Sciences* 106, 18004–18009. doi:10.1073/pnas.0904784106

FitzHugh, R. (1961). Impulses and physiological states in theoretical models of nerve membrane. *Biophysical Journal* 1, 445–466. doi:https://doi.org/10.1016/S0006-3495(61)86902-6

Hindmarsh, J. L., Rose, R. M., and Huxley, A. F. (1984). A model of neuronal bursting using three coupled first order differential equations. *Proceedings of the Royal Society of London. Series B. Biological Sciences* 221, 87–102. doi:10.1098/rspb.1984.0024

Izhikevich, E. M. (2007). *Dynamical systems in neuroscience* (MIT Press)

Maex, R. and Schutter, E. D. (1998). Synchronization of Golgi and Granule Cell Firing in a Detailed Network Model of the Cerebellar Granule Cell Layer. *Journal of Neurophysiology* 80, 2521–2537. doi:10.1152/jn.1998.80.5.2521. PMID: 9819260

Mainen, Z. F., Joerges, J., Huguenard, J. R., and Sejnowski, T. J. (1995). A model of spike initiation in neocortical pyramidal neurons. *Neuron* 15, 1427–1439. doi:10.1016/0896-6273(95)90020-9

Morris, C. and Lecar, H. (1981). Voltage oscillations in the barnacle giant muscle fiber. *Biophysical Journal* 35, 193–213. doi:https://doi.org/10.1016/S0006-3495(81)84782-0
